# A Toolbox for Representational Similarity Analysis

**DOI:** 10.1371/journal.pcbi.1003553

**Published:** 2014-04-17

**Authors:** Hamed Nili, Cai Wingfield, Alexander Walther, Li Su, William Marslen-Wilson, Nikolaus Kriegeskorte

**Affiliations:** 1 MRC Cognition and Brain Sciences Unit, Cambridge, United Kingdom; 2 Department of Computer Science, University of Bath, Bath, United Kingdom; 3 Department of Experimental Psychology, University of Cambridge, Cambridge, United Kingdom; UCSD, United States of America

## Abstract

Neuronal population codes are increasingly being investigated with multivariate pattern-information analyses. A key challenge is to use measured brain-activity patterns to test computational models of brain information processing. One approach to this problem is representational similarity analysis (RSA), which characterizes a representation in a brain or computational model by the distance matrix of the response patterns elicited by a set of stimuli. The representational distance matrix encapsulates what distinctions between stimuli are emphasized and what distinctions are de-emphasized in the representation. A model is tested by comparing the representational distance matrix it predicts to that of a measured brain region. RSA also enables us to compare representations between stages of processing within a given brain or model, between brain and behavioral data, and between individuals and species. Here, we introduce a Matlab toolbox for RSA. The toolbox supports an analysis approach that is simultaneously data- and hypothesis-driven. It is designed to help integrate a wide range of computational models into the analysis of multichannel brain-activity measurements as provided by modern functional imaging and neuronal recording techniques. Tools for visualization and inference enable the user to relate sets of models to sets of brain regions and to statistically test and compare the models using nonparametric inference methods. The toolbox supports searchlight-based RSA, to continuously map a measured brain volume in search of a neuronal population code with a specific geometry. Finally, we introduce the linear-discriminant *t* value as a measure of representational discriminability that bridges the gap between linear decoding analyses and RSA. In order to demonstrate the capabilities of the toolbox, we apply it to both simulated and real fMRI data. The key functions are equally applicable to other modalities of brain-activity measurement. The toolbox is freely available to the community under an open-source license agreement (http://www.mrc-cbu.cam.ac.uk/methods-and-resources/toolboxes/license/).

This is a *PLOS Computational Biology* Software Article

## Introduction

Brain science is constantly developing its techniques of brain-activity measurement. Neuronal recordings have always offered superior spatial and temporal resolution, and are improving in terms of the numbers of channels that can be recorded simultaneously in animal models. Functional magnetic resonance imaging (fMRI) has always had superior coverage and very large numbers of channels, enabling us to noninvasively measure the entire human brain simultaneously with tens to hundreds of thousands of voxels, and it has begun to invade the submillimeter range in humans. In the near future, it might be possible to image the activity of every cell within a functional area with millisecond temporal resolution [1].

A fundamental challenge is to use these rich spatiotemporal measurements to learn about brain information processing. Linear decoding analyses have helped reveal what information is present for linear readout in each region [2]–[11]. Beyond linear decoding, we would like to characterize neuronal population codes more comprehensively, including not only what information is present, but also the format, in which the information is represented. In addition, we would like to use activity measurements to test computational models of brain information processing [12]. One approach to these challenges is representational similarity analysis (RSA [13]; for a review of recent studies, see [14]).

In contrast to decoding analysis, which detects information about predefined stimulus categories in response patterns, RSA tests hypotheses about the representational geometry, which is characterized by the representational dissimilarities among the stimuli. RSA can relate brain activity patterns to continuous and categorical multivariate stimulus descriptions. When the stimulus description is the internal representation in a computational model, RSA can be used to test the model. RSA can also relate brain representations to behavioral data, such as similarity judgments e.g. [13], [15]–[16].

Although RSA has been successfully applied in many studies (e.g. [17]–[23] and many more reviewed in [14]), there has not been any freely available set of analysis tools implementing this method. An easy-to-use toolbox for RSA promises to help newcomers get started and could also provide a basis for collaborative development of further methodological advances across labs. Here we describe a freely available toolbox developed in Matlab. The toolbox provides a core set of functions for performing the data- and hypothesis-driven analyses of RSA. It includes a number of demo scripts that demonstrate key analyses based on simulated and real brain-activity data. These scripts come ready to run and provide an easy start for learning how to combine components to perform the desired analyses in Matlab. They may also serve as prototypes for a user's own analyses. The toolbox does not currently provide a graphical user interface; knowledge of Matlab is required. The outputs of the analyses are visualizations of brain representations and results of inferential analyses that test and compare alternative theoretical models. The toolbox supports a range or nonparametric frequentist inference techniques (signed-rank, randomization, and bootstrap tests), which are applicable to single subjects as well as groups of subjects, and can treat subjects and/or stimuli as random effects.

**Figure 1:**
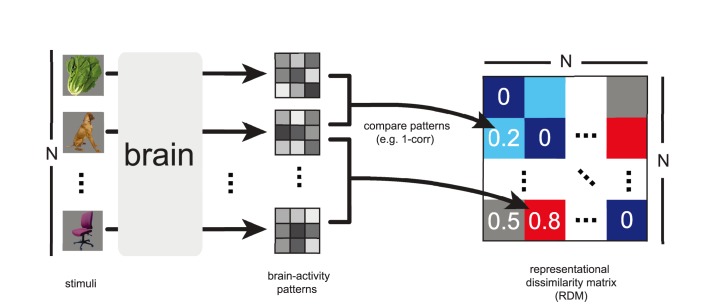
Computation of the representational dissimilarity matrix (RDM). During the experiment, each subject's brain activity is measured while the subject is exposed to N experimental conditions, such as the presentation of sensory stimuli. For each brain region of interest, an activity pattern is estimated for each experimental condition. For each pair of activity patterns, a dissimilarity is computed and entered into a matrix of representational dissimilarities. When a single set of response-pattern estimates is used, the RDM is symmetric about a diagonal of zeros. The dissimilarities between the activity patterns can be thought of as distances between points in the multivariate response space. An RDM describes the geometry of the representation and serves as a signature that can be compared between brains and models, between different brain regions, and between individuals and species.

**Figure 2:**
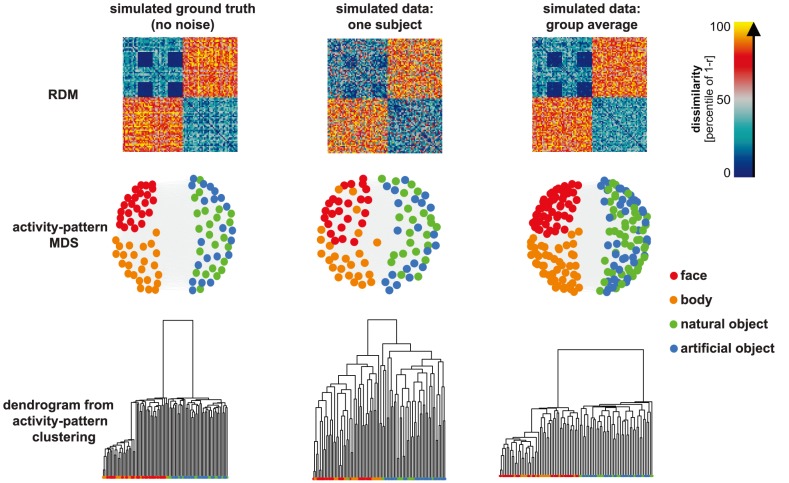
Visualizing representations as RDMs, 2D arrangements, and clustering dendrograms. Percentiled RDMs are displayed in the top row. The left RDM corresponds to the simulated ground truth (dissimilarities measured before adding noise). The middle RDM is an example of a simulated single-subject RDM (dissimilarities measured after adding isotropic Gaussian noise to the ground-truth patterns). The group-average RDM (right) is computed by averaging the RDMs for all 12 simulated subjects, which reduces the noise. Visual inspection reveals the simulated structure designed here to be similar to the human-IT RDM from Kriegeskorte et al. [19], with two main clusters corresponding to animate and inanimate objects and a cluster corresponding to human and animal faces. Two-dimensional arrangements (middle row, computed by MDS with metric stress criterion) provide a spatial visualization of the approximate geometry, without assuming any categorical structure. The third row displays the results of hierarchical agglomerative clustering to the three RDMs. Clustering starts with the assumption that there is some categorical structure and aims to reveal the categorical divisions. MDS plots and dendrograms share the same category color code (see color legend).

**Figure 3:**
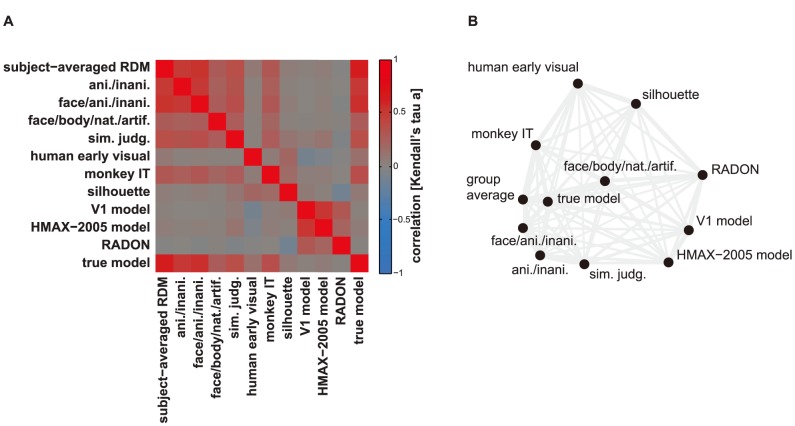
Visualizing the relationships among multiple representations. (**A**) Matrix of RDM correlations. Each entry compares two RDMs by Kendall's τ_A_. The matrix is symmetric about a diagonal of ones. (**B**) MDS of the RDMs. Each point represents an RDM, and distances between the points approximate the τ_A_ correlation distances (1 minus τ_A_) among the RDMs. The 2D distances are highly correlated (0.94, Pearson; 0.91, Spearman) with the RDM correlation distances. Visual inspection reveals that the group-average RDM is similar to the ground-truth RDM. However, the group-average RDM is also similar to some other model RDMs.

**Figure 4:**
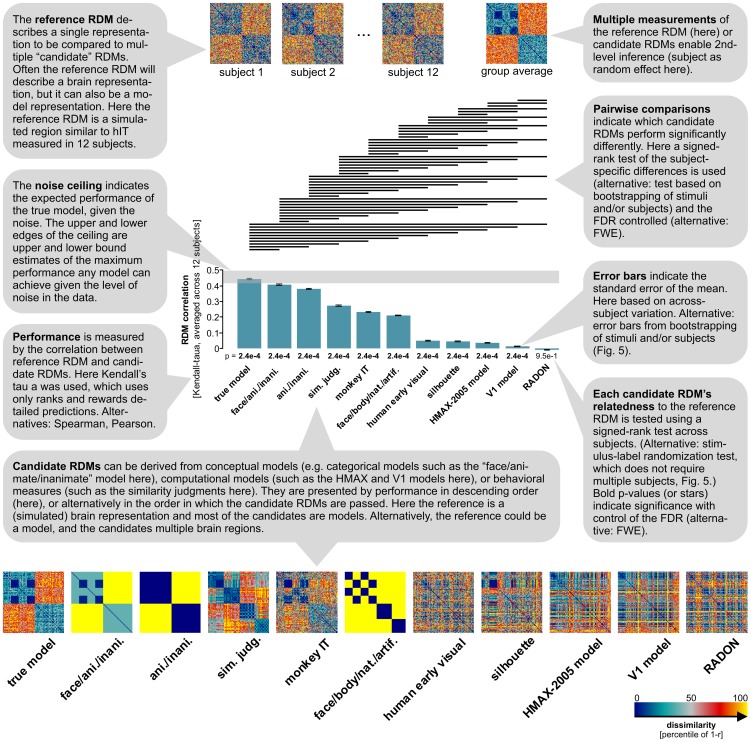
Simulated representation – inferential comparisons of multiple model representations. Several candidate RDMs are tested and compared for their ability to explain the reference RDM. As expected, the *true model* corresponding to the simulated ground truth (no noise) is the most similar candidate RDM to the reference. Note that the true model falls within the ceiling range, indicating that it performs as well as any possible model can, given the noise in the data. The second best fit among the candidate RDMs is the categorical model with some extra information about the within-animate category structure. This model reflects the categorical clustering in the simulated data, but misses the simulated within-category structure. A horizontal line over two bars indicates that the two models perform significantly differently. The pairwise statistical comparisons show that the true model is significantly better than all other candidate RDMs. Most of the other pairwise comparisons are significant as well, illustrating the power of the signed-rank test used for comparing candidate performances in this simulated scenario. Kendall's τ_A_ is used as a measure of RDM similarity, because candidates include categorical models (i.e. models predicting equal dissimilarities for many pairs of stimuli). Other rank-correlation coefficients overestimate the performance of categorical candidate RDMs (Figure S2 in Text S1). All candidate RDMs except that obtained from the RADON model are significantly related to the reference RDM (p values from one-sided signed-rank test across single-subject estimates beneath the bars).

We start with a brief description of the basic principles of the method and in particular the notion of a representational dissimilarity matrix (RDM), the core concept of RSA (Figure 1). We then proceed with a description of RSA in three steps. The steps are illustrated by applying the toolbox to simulated data (Figures 2–4). We then apply the key inferential analyses to real fMRI data (previously analyzed in [19]). This provides an example of how new biological insights can be gained from the technique by using the toolbox.

### Basics of representational similarity analysis

In studies of brain activity, subjects typically experience a number of experimental conditions while some correlate of neuronal activity is measured at multiple brain locations. In perceptual studies, the experimental conditions typically correspond to distinct stimuli. We will use the more specific term “stimulus” here for simplicity, with an understanding that the methods apply to non-perceptual (e.g. imagery or motor) experiments as well. The vector of activity amplitudes across response channels (i.e. voxels in fMRI, neurons or sites in cell recording) within a region of interest (ROI) is referred to as the *activity pattern*. Each stimulus is associated with an activity pattern, which is interpreted as the representation of the stimulus (or of the mental state associated with the experimental condition) within the ROI. Typically, the activity pattern is a spatial pattern. However, it may also be a spatiotemporal pattern [24]–[25].

RSA characterizes the representation in each brain region by a representational dissimilarity matrix (RDM, Figure 1). The most basic type of RDM is a square symmetric matrix, indexed by the stimuli horizontally and vertically (in the same order). The diagonal entries reflect comparisons between identical stimuli and are 0, by definition, in this type of RDM. Each off-diagonal value indicates the dissimilarity between the activity patterns associated with two different stimuli. The dissimilarities can be interpreted as distances in the multivariate response space. The RDM thus describes the geometry of the arrangement of patterns in this space. Popular distance measures are the correlation distance (1 minus the Pearson correlation, computed across voxels or sites of the two activity patterns), the Euclidean distance (the square root of the sum of squared differences between the two patterns), and the Mahalanobis distance (which is the Euclidean distance measured after linearly recoding the space so as to whiten the noise). The goal of RSA is to understand the representational geometry of a brain region. This is achieved by visualizing the representational distances in 2D and by statistically comparing the brain region's RDM to various model RDMs.

RDMs can be derived from a variety of sources beyond brain-activity patterns. For example, one can define an RDM on the basis of behavioral measures that capture the discriminability of different objects, such as judgments of dissimilarity, frequencies of confusions, or reaction times in a discrimination task. Hypotheses about the representations in a given brain region might also make specific predictions about their similarity structure. We may, for example, hypothesize that several stimuli should be represented as similar to each other because they share a semantic feature. This prediction can be expressed in an RDM. One may also obtain RDMs from computational models. For example, an RDM may be derived from the representation in a hidden layer of units in a neural network model. We refer to RDMs derived from either conceptual or computational models, or from behavioral data, as model RDMs.

### Design and implementation

The toolbox implements RSA in a stepwise manner. Its components can be used for analyzing dissimilarity matrices derived from any source. The input to the toolbox is the set of activity patterns corresponding to the experimental conditions for each ROI in each subject. In the first step, the brain-activity-based RDMs are computed and visualized. Descriptive visualizations give an intuitive sense of the representational geometry, revealing which pairs of stimuli are represented distinctly and which are represented similarly. In the second step, different RDMs are compared and the relationships among RDMs are visualized. This serves to reveal the extent to which the representational geometries in brain regions and models are similar to each other. These first two steps are descriptive and the visualizations will reflect both signals and noise, precluding any definite inferences. The third step is statistical inference on (a) the ability of each model RDM to account for each brain representation, and (b) the differences among models in their ability to account for each brain representation.

In order to demonstrate the three steps of analysis, we apply the toolbox to both simulated and real brain-activity data. Simulations enable us to define arbitrary hypothetical representational geometries. In a simulation, we know the “ground truth”, i.e. the noiseless true patterns underlying the noisy measurements that form the input to the analyses. This enables us to test how well our methods, despite the noise, can reveal the true representational geometry underlying the data.

The simulated data recreate an RDM similar to the one observed for human IT for a set of 92 images [19]. This RDM is characterized by two major clusters, corresponding to animate and inanimate objects (roughly the first and the second half of the set of 92 stimuli, respectively). Within the animates, there is a subcluster corresponding to faces. This cluster includes human and animal faces and appears as two small blue squares along the diagonal (corresponding to comparisons within human and within animal faces) and two small blue off-diagonal squares (corresponding to comparisons between human and animal faces). We first created a hypothetical ground-truth RDM (Figure 2, top left) by linearly combining the noisy estimate of the human-IT RDM from [19] with a categorical-model RDM. We then created a set of 92 patterns in a 100-dimensional response space, whose RDM matched the ground-truth RDM. (This was achieved by randomly sampling patterns from an isotropic Gaussian and then driving them to conform to the ground-truth RDM using forces.) Finally, we assumed the resulting patterns to be the “true” representation, and simulated data for 12 subjects, by adding a realistic level of isotropic Gaussian noise to the patterns.

## Results

**Figure 5:**
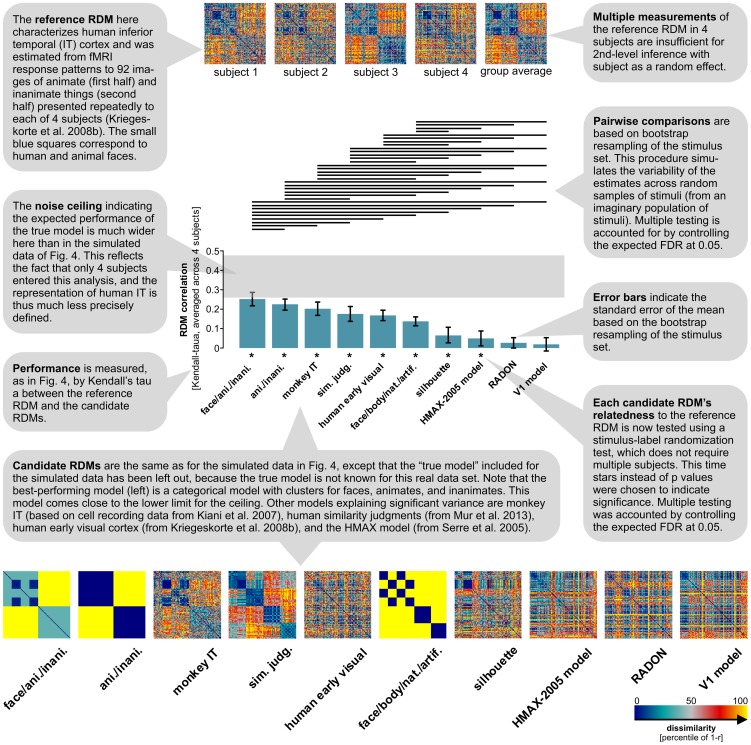
Human IT (real data) – inferential comparisons of multiple model representations. Like Fig. 4, this figure demonstrates inferential analyses supported by the toolbox. Here, however, inference is performed on real data from fMRI. The smaller number of subjects (4) precludes the use of second-level inference with subject as a random effect. Relatedness to the reference RDM is therefore tested using stimulus-label randomization and the pairwise performance comparisons among the candidate RDMs (along with the error bars) are based on bootstrap resampling of the stimulus set. The models are the same as in Fig. 4 and reproduced here for convenience (except for the “true model”, which is unknown for the real data). The comment bubbles detail the key changes in comparison to the analysis of Fig. 4, illustrating an alternative scenario for RSA statistical inference.

Figures 2–4 show the results of the basic steps of RSA for a simulated data set – along with the ground truth the analysis is meant to reveal. Figure 5 shows the application of the key final inferential analyses to real data (from [19]). A good way to get started with the toolbox is to run DEMO1_RSA_ROI_simulatedAndRealData.m, which reproduces all the results presented for simulated and real data in this paper (Figures 2–5, not including the additional results in Text S1).

### Step 1 — computing and visualizing RDMs

The first step is the calculation and visualization of the RDMs. This step is data-driven and helps reveal the dimensions of the stimulus space that are most strongly reflected in the response patterns. Figure 2 (top row) shows the RDMs for the simulated data. The group-average RDM better replicates the geometry simulated as ground truth, than the noisy single-subject RDMs. Multidimensional scaling [26]–[29] or t-SNE [30] may also be used in this step to visualize the similarity structure of the RDMs. These methods arrange the stimuli in a 2D plot such that the distances among them reflect the dissimilarities among the response patterns they elicited. Thus, stimuli that are placed closer together in these arrangements elicited more similar response patterns. Such visualizations provide an intuitive sense of the distinctions that are emphasized and de-emphasized by a population code. These methods are data-driven and do not presume a categorical structure. Hierarchical cluster trees (Figure 2, bottom) can help reveal categorical divisions. Unlike MDS, this technique assumes the existence of some categorical structure, but it does not assume any particular grouping into categories. In summary, step 1 consists in data-driven, exploratory methods that reveal the geometry of each representation by visualizing the RDMs and corresponding 2D arrangements and hierarchical cluster trees. These methods can be applied to each brain and model RDM. In step 2, the relationship among the representations in different brain regions and models will be explored.

### Step 2 — comparing brain and model RDMs

The second step of RSA is the descriptive visualization of the relationships among brain and model RDMs. To this end, we first consider the matrix of pairwise correlations between all brain and model RDMs. This matrix (Figure 3A) reveals, which representations in brain regions or models are similar, and which are dissimilar. Any metric that quantifies the extent to which two matrices are “in agreement” could be used as a measure of RDM similarity. We do not in general want to assume a linear relationship between the dissimilarities. Unless we are confident that our model captures not only the neuronal representational geometry but also its possibly nonlinear reflection in our response channels (e.g. fMRI patterns), assuming a linear relationship between model and brain RDMs appears questionable. We therefore prefer to assume that a model RDM predicts merely the rank order of the dissimilarities. For this reason we recommend the use of rank-correlations for comparing RDMs [19].

Classical rank correlation measures are Spearman's rank correlation coefficient (which is the Pearson correlation coefficient computed on ranks), Kendall's rank correlation coefficient τ_A_ (“tau a”, which is the proportion of pairs of values that are consistently ordered in both variables), and the closely related coefficients τ_B_, and τ_C_ (which deal with ties in different ways). We recommend Kendall's τ_A_, when comparing models that predict tied ranks to models that make more detailed predictions. Kendall's τ_A_ is more likely than τ_B_, τ_C_, and the Pearson and Spearman correlation coefficients to prefer the true model over a simplified model that predicts tied ranks for a subset of pairs of dissimilarities (Supplementary Figure S2). Note that Matlab's Kendall rank correlation function implements τ_B_, but the toolbox includes the more appropriate τ_A_. Unfortunately, τ_A_ takes much longer to compute than the Spearman correlation coefficient, which can slow down randomization and bootstrap inference (step 3) substantially for large RDMs. In the absence of models that predict tied ranks, the Spearman correlation coefficient is a good alternative.

Visual inspection of the correlation matrix of RDMs (Figure 3A) enables the user to get a sense of how similar the representations in different brain regions and models are to each other. The MDS plot based on this matrix (Figure 3B) provides an intuitive overview of the relationships among the brain and model RDMs. However, statistical inference on the RDMs (step 3) is required to draw definite conclusions about these relationships.

### Step 3 — statistical inference

In Step 3, the final step, we perform statistical inference to assess whether RDMs are related and whether there are differences in the degree of relatedness between RDMs. For example, we might want to test which of several models explain variance in a given brain representation and whether some of them explain the representation better than others. Alternatively, we might want to test for which of several brain representations a given model explains variance, and whether it explains some brain representations better than others. In either case, we are relating one RDM (called the *reference RDM*) to multiple other RDMs (called the *candidate RDMs*).

Figure 4 shows the results of statistical inference for our simulated data set. In this example, the reference RDM is a (simulated) brain RDM (to be explained) and the candidate RDMs are model RDMs (serving to explain). Note that we refer to the reference RDM as a single representation, even though the analysis is based on one reference-RDM estimate per subject. The relatedness of a candidate RDM to the reference RDM is measured as the average across subjects of the correlations between the candidate RDM and the single-subject reference-RDM estimates.

The relatedness of each candidate RDM to the reference RDM (Figure 4, bar height) was tested using a one-sided signed-rank test [31] across the single-subject RDM correlations (p values under bars). This is the default test in the toolbox when there are 12 or more subjects (see Figures 4 and 5 and Text S1, in particular Figure S1, for the full range of statistical tests and the default choices). Note that although several models have very small correlations with the simulated reference RDM here, all except the RADON model are significantly related to the reference RDM.

In order to test whether two candidate RDMs *differ* in their relatedness to the reference RDM, the toolbox computes the difference between the RDM correlations in each subject and performs a two-sided signed-rank test across subjects here. As before, this is the default test when there are 12 or more subjects. This procedure is repeated for each pair of candidate RDMs, yielding a large number of statistical comparisons. Multiple testing is accounted for by controlling the false-discovery rate (Benjamini and Hochberg, [32]; by default, alternative: familywise error rate). The significant comparisons are indicated by horizontal lines above the bars.

Note that the analysis in Figure 4 also includes other brain RDMs (monkey IT, based on [6]; human early visual cortex, based on [19]) among the candidate RDMs. Comparing brain RDMs to other brain RDMs can reveal the relationships between their representations (“representational connectivity” [13]). However, performance comparisons between candidate RDMs affected by noise to different degrees (such as noiseless models and brain RDMs) should not be formally interpreted.

Importantly, the bar graph includes an estimate of the noise ceiling. The noise ceiling is the expected RDM correlation achieved by the (unknown) true model, given the noise in the data. An estimate of the noise ceiling is important for assessing to what extent the failure of a model to reach an RDM correlation close to 1 is caused by a deficiency of the model or by the limitations of the experiments (e.g. high measurement noise and/or limited amount of data). If the best model does not reach the noise ceiling, we should seek a better model. If the best model reaches the noise ceiling, but the ceiling is far below 1, we should improve our experimental technique, so as to gain sensitivity to enable us to detect any remaining deficiencies of our model.

The noise ceiling is indicated by a gray horizontal bar, whose upper and lower edges correspond to upper- and lower-bound estimates on the group-average correlation with the RDM predicted by the unknown true model. Note that there is a hard upper limit to the average correlation with the single-subject reference-RDM estimates that any RDM can achieve for a given data set. Intuitively, the RDM maximizing the group-average correlation lies at the center of the cloud of single-subject RDM estimates. Where exactly this “central” RDM falls depends on the chosen correlation type. For the Pearson correlation, we first z-transform the single-subject RDMs. For the Spearman correlation, we rank-transform the RDMs. After this transformation, the squared Euclidean distance is proportional to the respective correlation distance. This motivates averaging of the single-subject RDMs to find the RDM that minimizes the average of the squared Euclidean distances and, thus, maximizes the average correlation (see Text S1 for the proof). For Kendall's τ_A_, we average the rank-transformed single-subject RDMs and use an iterative procedure to find the RDM that has the maximum average correlation to the single-subject RDMs.

The average RDM (computed after the appropriate transform for each correlation type) can be thought of as an estimate of the true model's RDM. This estimate is overfitted to the single-subject RDMs. Its average correlation with the latter therefore overestimates the true model's average correlation, thus providing an upper bound. To estimate a lower bound, we employ a leave-one-subject-out approach. We compute each single-subject RDM's correlation with the average of the other subjects' RDMs. This prevents overfitting and underestimates the true model's average correlation because the amount of data is limited, thus providing a lower bound on the ceiling.

Figures 2–4 demonstrated the toolbox on simulated data, where the ground truth was known. In Figure 5, the inferential analyses are applied to a real data set (human IT, based on fMRI data from [19]). The structure of the reference RDM is very similar in the simulated and real data. However, we only have 4 subjects and so subject cannot be treated as a random effect in this analysis. The toolbox therefore uses a stimulus-label randomization test [33]–[34] to test the relatedness of each candidate RDM to the reference RDM, and a bootstrap test [35], based on resampling with replacement of the stimulus set, to compare the performance of different candidate RDMs.

## Additional Analysis Options

### Searchlight representational similarity analysis

ROI-based RSA analyzes the representational geometry in a predefined set of brain regions. However, other brain regions might also have representational geometries that conform to the predictions of our models. Searchlight analysis [5] provides a method of continuously mapping pattern information throughout the entire measured volume. The toolbox includes searchlight RSA [22] for fMRI data. RSA is carried out for a spherical cluster of voxels centered at each voxel. This provides an RDM-correlation map for each model RDM, which reveals where in the brain the local representation conforms to the model's predictions. Inference is performed at each voxel by a signed-rank test across subjects and the resulting p map is thresholded to control the false-discovery rate (see Text S1, in particular Figure S3, for details).

### The linear-discriminant t value: Combining the advantages of linear classifiers and representational similarity analysis

Linear classifiers have been successfully applied to a variety of neurophysiological data [2]–[4], [36]–[37, for an introduction see 9]. They find optimal weights and enable highly sensitive detection of distributed information in a population code that can be linearly read out. However, they reflect only categorical distinctions and do not characterize the representational geometry as richly as RSA does. This raises the question of whether the advantages of these methods can be combined. RDMs are distance matrices whose entries reflect the separation in the representation of each pair of stimuli. We could use a linear classifier to estimate the discriminability of each pair of stimuli, and interpret these discriminabilities as our distances. Here we introduce a new measure of separability for RSA that is based on linear discriminant analysis. We first divide the data into two independent sets. For each pair of stimuli, we then fit a Fisher linear discriminant to one set, project the other set onto that discriminant dimension, and compute the t value reflecting the discriminability between the two stimuli. We call this multivariate separation measure the linear-discriminant *t* (LD-*t*) value. It can be interpreted as a crossvalidated, normalized variation on the Mahalanobis distance (see Figure S4). Note, however, that it is not a distance in the mathematical sense, because it can be negative. The LD-*t* has a number of desirable properties. First, whereas distance measures are positively biased, it is symmetrically distributed around 0 (*t* distribution) when the true distance is 0. The LD-*t* therefore enables instant inference on the discriminability (by converting the *t* values to *p* values) for each pair of stimuli. Second, it enables inference on mean discriminabilities across many pairs of stimuli by within-subject randomization of stimulus labels or across-subjects random-effects tests. (Other distance measures require bias correction, e.g. subtracting an estimate of the expected distance for repetitions of the same stimulus.) Third, the LD-*t* works well for condition-rich designs, in which we have few trials (or even just one trial) for each particular stimulus. (We can obtain an error covariance estimate pooled over all stimuli, whereas a linear support vector machine fitted to a pair of response patterns would reduce to a minimum Euclidean-distance classifier.) Fourth, in contrast to decoding accuracy (which could also be computed for each stimulus pair), the LD-*t* is a continuous measure in each subject and does not suffer from a ceiling effect. (When the decoding accuracy is at its 100% ceiling, the LD-*t* still continuously reflects the separation of the patterns in the multivariate response space.) The LD-*t* is supported by the toolbox and its application illustrated in Figure S5 (see Text S1 for more details).

## Discussion

We introduced a toolbox for RSA that supports the analysis of representational dissimilarity matrices characterizing brain regions and models. First, the RDMs for brain regions and models and their inter-relationships are visualized. Then statistical inference is performed to decide what models explain significant variance and whether the models perform significantly differently. The toolbox additionally supports searchlight RSA, i.e. the continuous mapping of RSA statistics throughout the brain. Finally, we introduced the linear-discriminant *t* value as a measure of multivariate discriminability that bridges the gap between classifier decoding and RSA.

### Choosing the most appropriate statistical inference procedure

The toolbox uses frequentist nonparametric inference procedures. For testing the relatedness of two RDMs, the preferred (and default) method is the signed-rank test across subjects. This test provides valid inference and treats the variation across subjects as a random effect, thus supporting inference to the population. The toolbox requires that RDMs for 12 or more subjects are available. (The test could also be used for within-subject inference, if 12 or more independent RDM estimates from the same subject were available.) The fixed-effects alternative is to test RDM relatedness using the stimulus-label randomization test [13]. This test is definitely valid and expected to be more powerful than the signed-rank test across subjects, because it tests a less ambitious hypothesis: that the RDMs are related in the experimental group of subjects, rather than in the population. The stimulus-label randomization test can be used for a single subject or a group of any size. However, it does require a sufficient number of stimuli: at least 7, because for 6, there are only 6! = 720 unique permutations. The signed-rank test across subjects would work with as few as 4 stimuli (generating 6 dissimilarities, enough for rank correlations to take on an acceptable number of distinct values). However, the inference procedures have not been validated for very small numbers of conditions, so the toolbox currently requires 20 or more stimuli for the stimulus-label randomization test, and we suggest having at least 6 stimuli when using the signed-rank test across subjects. Note also that RSA lends itself to condition-rich designs and, in general, it is desirable to sample the stimulus space richly.

The relatedness of two RDMs can also be tested by bootstrapping the stimulus set and/or the subjects set. The motivation for bootstrapping is to simulate repeated sampling from the population. Bootstrapping, thus, can help generalize from the sampled subjects and/or stimuli to the population of subjects and/or the population of stimuli. (The population of stimuli would be a typically very large set of possible stimuli, of which the experimental stimuli can be considered a random sample.) However, the bootstrap might not provide a very realistic simulation of repeated sampling from the population. The basic bootstrap tests implemented in the toolbox are known to be slightly optimistic. Future extensions might include bias-corrected and accelerated bootstrap methods [35].

Similar considerations apply to the tests of difference between candidate RDMs regarding their relatedness to the reference RDM. Again, the preferred (and default) test is the signed-rank test across subjects, which supports generalization to the population. Stimulus-label randomization is not appropriate in this context, because it simulates the null hypothesis that the RDMs are unrelated (and the stimulus labels, thus, exchangeable), rather than the appropriate null hypothesis that both candidate RDMs are equally related to the reference RDM. The alternative to the signed-rank test is the bootstrap test. Again, this can be based on resampling of the subjects and/or the stimuli. The slight optimism of basic bootstrap tests should be kept in mind. However, at conservative thresholds and with correction for multiple testing, this test provides a reasonable alternative to the signed-rank test, when there are not enough subjects.

### Testing many models

A key feature of the toolbox is the statistical comparison of multiple models. Figures 4 and 5 illustrate a typical scenario, in which a wide range of qualitatively different models explain significant variance in a brain region's representational geometry. These models include categorical models, models based on simple image features, complex computational models motivated by neurophysiological findings, and behavioral models. The finding that a model explains some variance in a brain representation (or conversely allows above-chance-level decoding) reveals that the region contains the information the model represents. However, this is a very low bar for a computational account of a brain representation. Many models will explain some component of the variance, so finding one such model does not substantially advance our understanding of brain function. Theoretical progress requires that we compare multiple models [14]. The toolbox enables the user to find the best among a whole range of models, and to assess which other models it significantly outperforms. Importantly, the noise ceiling reveals whether a model fully accounts for the non-noise variance in the data, or leaves some variance to be explained.

If a computational model has parameters, these could be fitted with a separate data set (comprised of an independent sample of stimuli). Alternatively, if the parameter space is low-dimensional, it could be grid-sampled and all resulting RDM predictions entered as candidate RDMs for statistical comparison. Future extensions of the toolbox might include functions that support the fitting of parametric models and their validation with an independent data set.

### Relation to univariate encoding models

Univariate encoding models provide an alternative to RSA for testing computational models of brain information processing [38]–[41]. Both approaches test forward models, i.e. models that operate in the direction of information flow in the brain: from stimuli to brain responses. The shared aim is to test to what extent each model can account for the neuronal representation in a brain region. However, univariate encoding models are fitted to predict each response channel (e.g. each voxel in fMRI) separately. RSA, in contrast, compares the model representation to the brain representation at the level of the response pattern dissimilarities. The two approaches have complementary advantages. Predicting every response channel separately enables us to create a map of the intrinsic spatial organization for each brain region. Predicting the dissimilarities of multivariate response patterns abstracts from the single representational units and focuses on the population representational geometry. We lose the detailed spatial organization (the trees) and gain a population summary (the forest). The representational dissimilarity trick [14] enables us to test computational models without first having to fit a linear model using a separate data set of responses to an independent stimulus sample. It also enables uncomplicated tests of categorical and behavioral models and of relationships between brain regions and between individuals and species [19]. The parallels and differences between these two approaches have been explored in greater detail in [10].

## Availability and Future Directions

The toolbox is freely available to the community. The user can download the toolbox at http://www.mrc-cbu.cam.ac.uk/methods-and-resources/toolboxes/license/. The zip file containing the toolbox (rsatoolbox.zip, software S1) is also included in the supplementary materials.

There are a number of directions in which the toolbox might be extended in the future. First, we plan to add functionality for time-resolved RSA, including temporal-sliding-window techniques for electrophysiological data (MEG/EEG and invasive recordings). Such analyses can reveal the emergence and dynamics of representational geometries over the course of tens to hundreds of milliseconds after stimulus onset, reflecting recurrent neuronal computations [25], [42]. Second, we would like to include additional methods for characterizing representational geometries. For example, Diedrichsen et al. [43] have proposed a technique for decomposition of the pattern variance into components reflecting different stimulus-related effects and noise. This approach promises estimates of the representational geometry that are more comparable between representations affected by different levels of noise. Another relevant recent technique is kernel analysis, which can reveal the complexity of categorical boundaries in a representation [44]–[45]. We expect that the field will develop a range of such useful descriptive measures for representational geometries. These should be included in the toolbox. Finally, it would be desirable to complement the frequentist approach described here by Bayesian inference procedures. By sharing the toolbox with the community, we hope to accelerate the collaborative pursuit of these methodological directions, in addition to contributing to neuroscientific studies that aim to reveal the nature of representational geometries throughout the brain.

## Supporting Information

Figure S1 **Decision process for selection of statistical tests.** The flow diagram above shows the default decision process by which the statistical inference procedures are chosen in the toolbox. The analyses in Figures 4 and 5 of the paper correspond to paths in the flowchart that lead to the leftmost (simulation in Figure 4) and second from right (real data in Figure 5) box at the bottom. Note that the flowchart does not capture all possibilities. For example, the fixed-effects condition-label randomization test of RDM relatedness can be explicitly requested, even when there are 12 or more subjects' estimates of the reference RDM and the random-effects signed-rank test would be chosen by default.
(EPS)

Figure S2 **Spearman versus Kendall's τ_A_ rank correlation for comparing RDMs.** Here the inferential results from the paper using Kendall's τ_A_ (Figures 4, 5) are presented again (panels A, B), and compared to the results obtained using the Spearman correlation (panels C, D). The two rank correlation coefficients differ in the way they treat categorical models (blue bars) that predict tied dissimilarities. (**A**) For the simulated data, Kendall's τ_A_ correctly reveals that the true model (red bar) best explains the data. It is the only model that reaches the ceiling range, and it outperforms every other candidate significantly (horizontal lines above the bars). (**C**) For the Spearman correlation, the true model no longer has the greatest average correlation to the reference RDM. Two categorical candidate RDMs appear to outperform the true model, and significantly so (horizontal lines). Both of these categorical models and the true model now fall in the ceiling range. (**B, D**) For the real data, as well, categorical models (blue) are favored by the Spearman correlation.
(EPS)

Figure S3 **Group-level results for 20 simulated subjects.** (**A**) A representational geometry of 64 patterns falling into two clusters was simulated in a brain region (shown in green) in each of 20 subjects. Data outside the green region was spatially and temporally correlated noise (typical of fMRI data) with no design-related effects. Searchlight maps (searchlight radius = 7 mm) were generated by computing the correlation between a model RDM (reflecting the true cluster structure of the simulated patterns) and the searchlight RDM at each voxel in each subject. (**B**) At each voxel, a one-sided signed-rank test was applied to the subject-specific correlation values. The 3D map of *p* value was thresholded so as to control the expected false-discovery rate at 0.05. Voxels exceeding the threshold are highlighted (yellow). The maps in both panels are superimposed on an anatomical T1 image re-sliced to fit the simulated brain dimensions. The red contours depict the borders of the brain mask. RDMs were computed for searchlights centered on each voxel within the brain mask.
(EPS)

Figure S4 **Relationship between the linear-discriminant** ***t*** **value and the Mahalanobis distance.** In the Mahalanobis distance, the inverse of the error covariance (Σ) is pre- and post-multiplied by the difference vector between the pattern estimates (p1 and p2). If we use pattern estimates from an independent dataset (dataset 2) for the post-multiplication, we obtain the dataset-2 contrast estimate on the Fisher linear discriminant fit with dataset 1. This is because the first part of the definition of the Mahalanobis distance equals the weight vector w of the Fisher linear discriminant. The LD-*t* is the Fisher linear discriminant contrast (as shown) normalized by its standard error (estimated from the residuals of dataset 2 after projection on the discriminant dimension).
(TIF)

Figure S5 **Random-effects inference on LD-** ***t*** **RDMs.** (**A**) Two fMRI datasets were simulated for 20 subjects. We simulated fMRI time-course data Y based on a realistic fMRI design matrix (X) with hemodynamic response predictors for 64 stimuli and patterns (B) with a predefined hierarchical cluster structure (two categories, each comprising two subcategories). The simulated data were Y = XB+E, where E is the time-by-response errors matrix, consisting of Gaussian noise temporally and spatially smoothed by convolution with Gaussians to create realistic degrees of temporal and spatial autocorrelation. The LD-*t* RDMs were computed for each subject and averaged across subjects. The group-average LD-*t* RDM is shown using a percentile color code. (**B**) Inference on LD-*t* RDMs with subject as random effect. LD-*t* analysis can serve the same purpose as classifier decoding analysis, to test for pattern information discriminating two stimuli. For each pair of stimuli, we used a one-sided signed-rank test across subjects and obtained a *p* value. The left panel shows the pairs with p<0.05, uncorrected (red). The middle panel shows the pairs that survive control of the expected false-discovery rate (q<0.05). The right panel shows the pairs that survive Bonferroni correction (p<0.05, corrected).
(EPS)

Software S1 **The zip file contains the complete RSA toolbox.** It also contains demo functions and brain-activity- and behavior-based representational dissimilarity matrices used by the demo functions. DEMO1_RSA_ROI_simulatedAndRealData.m reproduces the main parts of figures 2–5 of the main paper. The toolbox is written in Matlab and requires the Matlab programming environment.
(ZIP)

Text S1 **The supplementary materials (additional text) for the manuscript.**
(DOCX)
