# Supplementary figures and images for: A Toolbox for Representational Similarity Analysis
Source: PLoS Comput Biol. 2014 Apr 17;10(4):e1003553. doi: 10.1371/journal.pcbi.1003553 (PMC3990488; doi:10.1371/journal.pcbi.1003553)

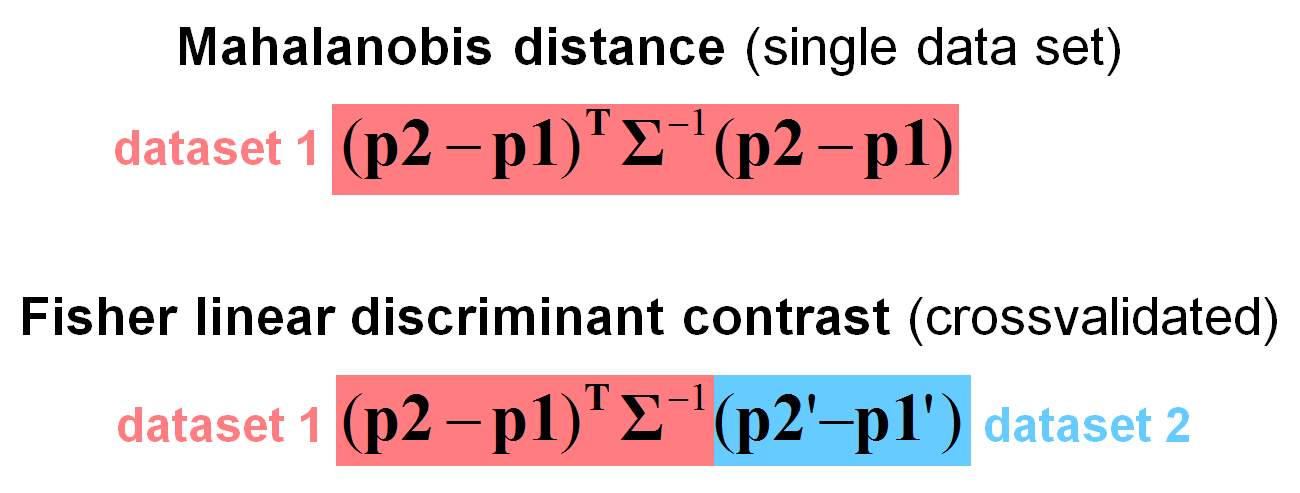

Supplement: Figure S4 — Relationship between the linear-discriminant t value and the Mahalanobis distance. In the Mahalanobis distance, the inverse of the error covariance (Σ) is pre- and post-multiplied by the difference vector between the pattern estimates (p1 and p2). If we use pattern estimates from an independent dataset (dataset 2) for the post-multiplication, we obtain the dataset-2 contrast estimate on the Fisher linear discriminant fit with dataset 1. This is because the first part of the definition of the Mahalanobis distance equals the weight vector w of the Fisher linear discriminant. The LD-t is the Fisher linear discriminant contrast (as shown) normalized by its standard error (estimated from the residuals of dataset 2 after projection on the discriminant dimension). (TIF) [file pcbi.1003553.s004.tif]
