# Supplementary material for: A Toolbox for Representational Similarity Analysis
Source: PLoS Comput Biol. 2014 Apr 17;10(4):e1003553. doi: 10.1371/journal.pcbi.1003553 (PMC3990488; doi:10.1371/journal.pcbi.1003553)
Supplement: Software S1 — The zip file contains the complete RSA toolbox. It also contains demo functions and brain-activity- and behavior-based representational dissimilarity matrices used by the demo functions. DEMO1_RSA_ROI_simulatedAndRealData.m reproduces the main parts of figures 2–5 of the main paper. The toolbox is written in Matlab and requires the Matlab programming environment. (ZIP) [file pcbi.1003553.s006.zip › rsatoolbox/Demos/compareRefRDM2candRDMs_barGraph.pdf]

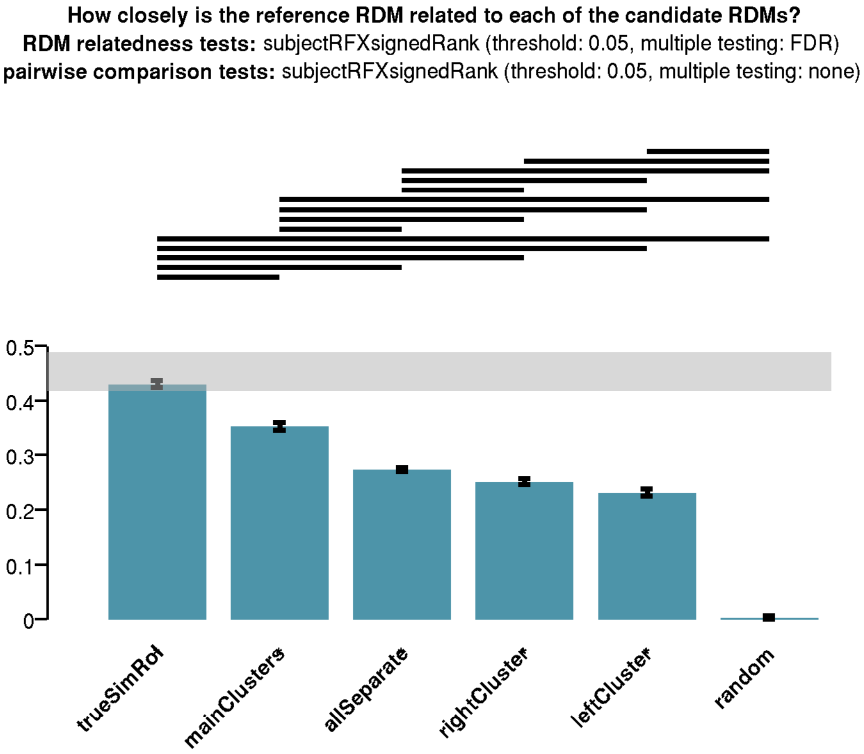
How closely is the reference RDM related to each of the candidate RDMs?
RDM relatedness tests: subjectRFXsignedRank (threshold: 0.05, multiple testing: FDR)
pairwise comparison tests: subjectRFXsignedRank (threshold: 0.05, multiple testing: none)
0.5
0.4
0.3
0.2
0.1
0
trueSimRot
mainClusters
allSeparate
rightCluster
leftCluster
random
