# Supplementary material for: A Toolbox for Representational Similarity Analysis
Source: PLoS Comput Biol. 2014 Apr 17;10(4):e1003553. doi: 10.1371/journal.pcbi.1003553 (PMC3990488; doi:10.1371/journal.pcbi.1003553)
Supplement: Software S1 — The zip file contains the complete RSA toolbox. It also contains demo functions and brain-activity- and behavior-based representational dissimilarity matrices used by the demo functions. DEMO1_RSA_ROI_simulatedAndRealData.m reproduces the main parts of figures 2–5 of the main paper. The toolbox is written in Matlab and requires the Matlab programming environment. (ZIP) [file pcbi.1003553.s006.zip › rsatoolbox/Demos/compareRefRDM2candRDMs_RDMcomparisonPvalues.pdf]

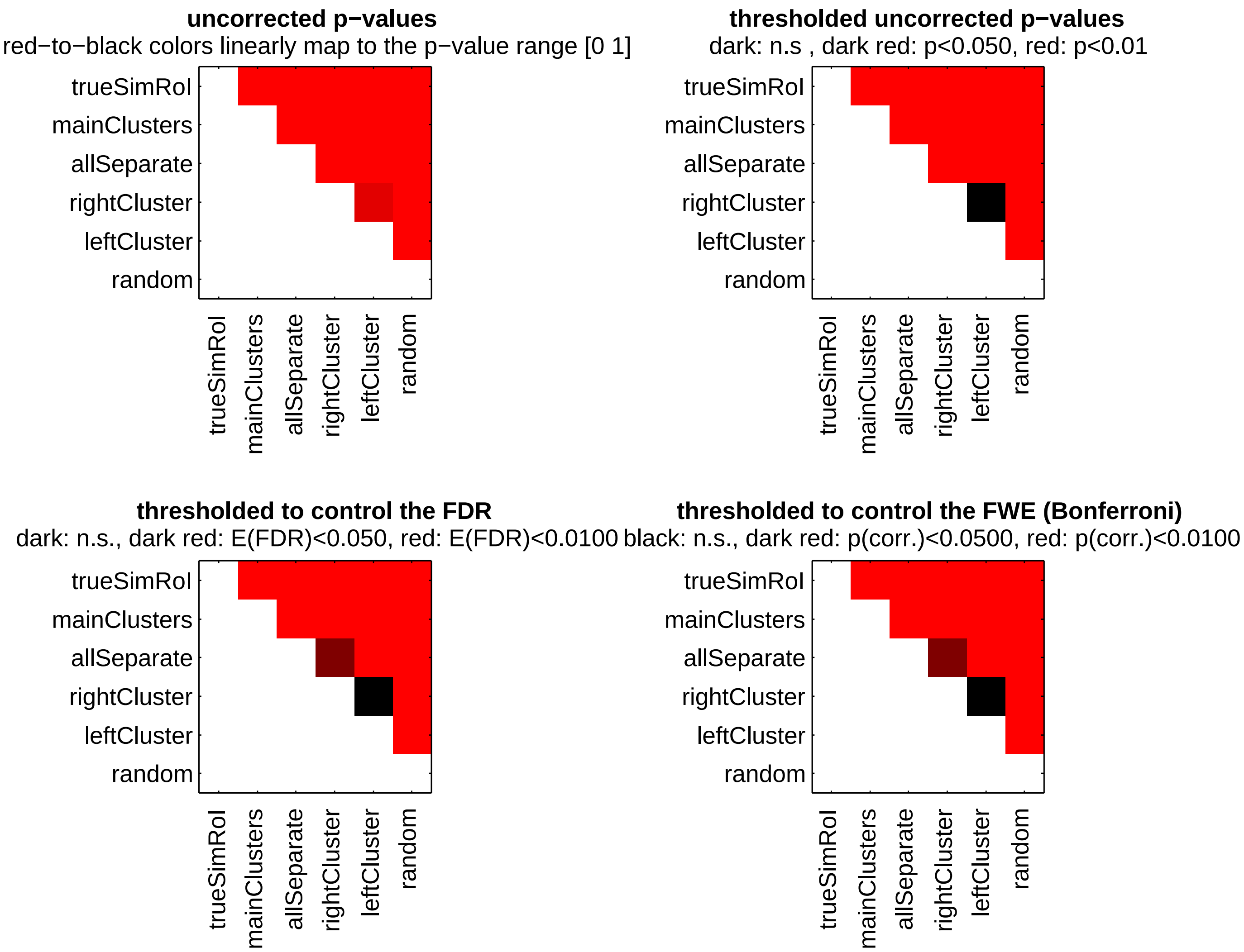

uncorrected p−values
red−to−black colors linearly map to the p−value range [0 1]
thresholded uncorrected p−values
dark: n.s , dark red: p<0.050, red: p<0.01
thresholded to control the FDR
dark: n.s., dark red: E(FDR)<0.050, red: E(FDR)<0.0100
thresholded to control the FWE (Bonferroni)
black: n.s., dark red: p(corr.)<0.0500, red: p(corr.)<0.0100
trueSimRoI
mainClusters
allSeparate
rightCluster
leftCluster
random
