# Supplementary material for: A Toolbox for Representational Similarity Analysis
Source: PLoS Comput Biol. 2014 Apr 17;10(4):e1003553. doi: 10.1371/journal.pcbi.1003553 (PMC3990488; doi:10.1371/journal.pcbi.1003553)
Supplement: Software S1 — The zip file contains the complete RSA toolbox. It also contains demo functions and brain-activity- and behavior-based representational dissimilarity matrices used by the demo functions. DEMO1_RSA_ROI_simulatedAndRealData.m reproduces the main parts of figures 2–5 of the main paper. The toolbox is written in Matlab and requires the Matlab programming environment. (ZIP) [file pcbi.1003553.s006.zip › rsatoolbox/Documentation/toolbox documentation.pdf]

REPRESENTATIONAL SIMILARITY ANALYSIS TOOLBOX

DOCUMENTATION

TABLE OF CONTENTS

Table of contents 1

Getting started with the toolbox 2

Formats for representational dissimilarity matrices 3

Applying the toolbox to collected fMRI data using the ‘recipes’ 4

Toolbox modules 6

Toolbox engines 20

Toolbox code dependencies 55

GETTING STARTED WITH THE TOOLBOX

The toolbox folder has a number of built-in, ready-to-use demo files that can serve as a prototype for the user's analysis. To run the demos and get figures similar to those in the paper, the user should proceed as follows:

(1) Download the toolbox from here: http://www.mrc-cbu.cam.ac.uk/methods-and-resources/toolboxes/license/

(2) Save a local copy of the RSAtoolbox folder.

(3) Open Matlab and set the current directory to the *Demo* subfolder of the toolbox folder (..\RSAtoolbox\Demo)

   a. Run `DEMO1_RSA_ROI_simulatedAndRealData.m` for a demonstration of ROI-based analysis using the toolbox. It simulates RDMs, analyzes them with RSA, and reproduces Figures 2-4.

   b. Run `DEMO2_RSA_ROI_sim.m` for a demonstration of the ROI-based RSA on simulated fMRI data. This script will familiarize the user with the pipeline for analyzing fMRI data.

   c. Run `DEMO3_LDt_sim.m` for a demonstration of the computation of LD-*t* RDMs and associated inference procedures. Running the script reproduces Figure S5.

   d. Run `DEMO4_RSAsearchlight_sim.m` for a demonstration of the searchlight analysis on simulated fMRI data. Running this script reproduces Figure S3.

The first three demos take a few minutes to run. The searchlight demo can take hours the first time it is run, because it needs to simulate data for the whole brain in multiple subjects. The searchlight analysis of the simulated data takes only a couple of minutes per subject on a modern workstation.

FORMATS FOR REPRESENTATIONAL DISSIMILARITY MATRICES

A representational dissimilarity matrix (RDM) can be stored in multiple formats that have different advantages and disadvantages. The RSA toolbox functions therefore handle several formats and generally automatically detect the format of the RDMs that are passed as arguments. The RDM formats and conversions between them are illustrated in Figure 1, below.

The simplest and most intuitive format for an RDM is a square matrix (with redundant entries in the upper and lower triangular regions and zeros along the diagonal). A more compact representation is the lower-triangular-vector format (`_ltv`). This format is used extensively by the toolbox and also by Matlab's pdist and related functions. The toolbox enables conversion between square and lower-triangular-vector formats via the functions `squareRDMs` and `vectorizeRDMs`.

Independently of the square/vector distinction, an RDM can be either just that matrix or vector, or a structure (Matlab struct) that additionally contains a name for the RDM and a colour. An RDM in the latter format with the meta-information included is called a 'wrapped' RDM. The names and colours are useful when jointly analysing a set of RDMs with different methods. Toolbox functions will then use appropriate colours and labels consistently across different analyses. The toolbox enables conversion of a simple-matrix or vector RDM into a wrapped RDM using the `wrapRDMs` and `wrapAndNameRDMs` functions. You can extract RDM matrices from structured RDM representations by using the `unwrapRDMs` function.

Finally, we often want to represent a set of RDMs in a single variable. If the RDMs are wrapped, then the toolbox uses a structured array. If the RDMs are simple matrices or lower-triangular vectors, then they are stacked along the third dimension. (Even vectors are stacked along the third to avoid ambiguity: a square matrix could otherwise be mistaken for a stack of vectors.) Multiple RDMs can be concatenated with the `concatRDMs` function to make an RDM set.

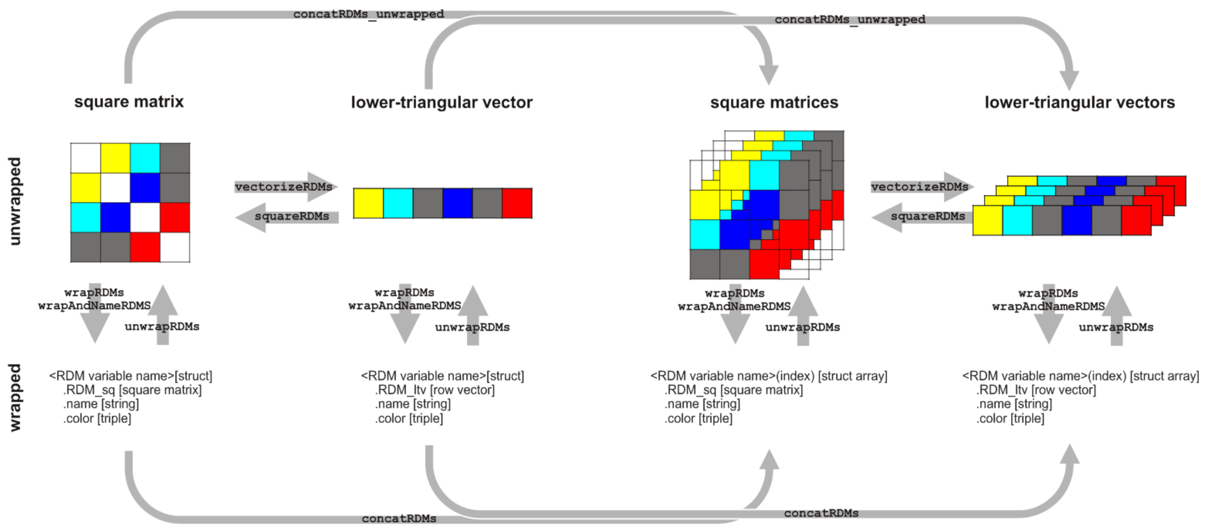

Figure 1: Alternative formats for representational dissimilarity matrices and the conversions between them.

APPLYING THE TOOLBOX TO COLLECTED FMRI DATA USING THE 'RECIPES'

We provide a recipe for region of interest analysis of fMRI data. This script reads the imaging data from different ROIs of different subjects and computes an RDM from each. It then combines the data and performs statistical inference.

It should be noted that users may need to change the settings specified here depending on the type of inference or visualization they require.

Here we explain the general information a user needs to know. Please refer to the previous sections for detailed description of different scripts that are employed.

Data preparation

This section prepares the data for further analysis. It is important to note that the toolbox does NOT perform pre-processing of the data and the user needs to take use of the other software packages (e.g. SPM) that are available and can serve the purpose.

The data needed for the analysis should be brain responses to all experimental conditions. These could either be Matlab (.mat) files or SPM (.img) files. The user has to provide the path to the full brain volumes per condition. This is where they have to change the `betaCorrespondence.m`. Only if they want to use SPM's beta images as the response patterns for RSA they can omit this part and put 'SPM' in place of `betaCorrespondence.m`

However in most cases (e.g. when the user wants to use the spmT images that are resulted from comparing each condition with the baseline) the user needs to use the `betaCorrespondence.m` and modify it.

RDM calculation

In this part of the script, RDMs are calculated for each subject and each region of interest. Also the model RDMs are converted into structures that could be handled by the toolbox. Furthermore RDMs would be averaged across subjects and/or sessions to obtain group-level representatives for each region of interest.

First-order visualization

This is essentially step 1. In this step, average RDMs are displayed first. (RDMs are averaged according to user's preferences, defaulting to subject- and session-averaged RDMs for each RoI.) Then the MDS plots and the dendrograms will be displayed and saved.

Relationship amongst multiple RDMs

Codes in this section implement step 2 of the analysis. This includes running the two modules called `pairwiseCorrelateRDMs` and `MDSRDMs`.

Statistical inference

The user is strongly recommended to read the section on compareRefRDM2candRDMs, the key function for statistical inference.

TOOLBOX MODULES

The toolbox has a number of modules. These modules that are listed in table S1 are the key functions of the toolbox.

constructRDMs

`[RDMs =] constructRDMs(responsePatterns, betaCorrespondence, userOptions)`

`constructRDMs` is a function which takes a matrix of imaging data and computes RDMs from it, one RDM for each ROI, subject, and scanning session. The RDMs are returned in a structure with names reflecting the ROI, subject and session. The returned RDMs are also saved. The RDM structure format ensures that the RDMs will be properly labeled in subsequent analyses.

`responsePatterns`: The input response patterns. It contains the masked brain activity maps in a structure such that `responsePatterns.(mask).(subject)` is a [`nMaskedVoxels nConditions nSessions`] matrix, where mask is a binary brain map that defines the ROI.

`betaCorrespondence`: The array of beta filenames. `betas(condition, session).identifier` is a string which refers to the filename (not including the path) of the SPM beta image. Alternatively, this can be the string "`SPM`", in which case the SPM metadata will be used to infer this information, provided that `userOptions.conditionLabels` is set, and the condition labels are the same as those used in SPM.

It must be noted that this module assumes that data is in such a format that the input structure has one field per subject, session and mask. Defining betaCorrespondence or choosing ‘`SPM`’ does not automatically compute the `responsePatterns` structure. At this point only the number of sessions and/or conditions are extracted from SPM or `betaCorrespondence`.

`userOptions`: The standard options structure containing the following fields:

`userOptions.analysisName`: A string which is prepended to the saved files.

`userOptions.rootPath`: A string describing the root path where files will be saved (inside created directories).

`userOptions.maskNames`: A cell array containing strings identifying the mask names. It defaults to the field names of the first subject of `responsePatterns`.

`userOptions.subjectNames`: A cell array containing strings identifying the subject names. Defaults to the field names in (the first mask of) `responsePatterns`.

`userOptions.distance`: A string indicating the distance measure with which to calculate the RDMs. Defaults to “Correlation”, but can be set to any Matlab distance measure.

`userOptions.RoIColor`: A triple indicating the [R G B] value of the colour which should be used to indicated RoI RDMs on various diagrams. Defaults to black ([0 0 0]).

The following files are saved by this function:

`userOptions.rootPath/RDMs/`

`userOptions.analysisName_RDMs.mat`

Contains a structure of ROI RDMs which is of size

`[nMasks, nSubjects, nSessions]` and with fields:, `RDM, name, color.`

`userOptions.rootPath/Details/`

`userOptions.analysisName_constructRDMs_Details.mat` is a file that contains the userOptions for this execution of the function and a timestamp.

figureRDMs

`figureRDMs(RDMs, userOptions[, localOptions])`

This is a function, which accepts a multidimensional structure of RDMs, puts them into a 1D structure, and then shows them.

`RDMs`: A structure of RDMs.

All RDMs in here will be concatenated and displayed.

`userOptions`: The options structure.

`userOptions.analysisName: A` string which is prepended to the saved files.

`userOptions.rootPath: A` string describing the root path where files will be saved (inside created directories).

`userOptions.saveFigurePDF: A` Boolean value. If true, the figure is saved as a PDF. Defaults to false.

`userOptions.saveFigurePS: A` Boolean value. If true, the figure is saved as a PS. Defaults to false.

`userOptions.saveFigureFig: A` Boolean value. If true, the figure is saved as a MATLAB .fig file. Defaults to false.

`userOptions.displayFigures: A B`oolean value. If true, the figure remains open after it is created. Defaults to true.

`userOptions.imagelables: D`efaults to empty (no image labels).

`userOptions.rankTransform`: Boolean value. If true, values in each RDM are separately transformed to lie uniformly in [0,1] with ranks preserved. If false, true values are represented. Defaults to true. This is for display only.

`localOptions`: Further options specific to this function.

`localOptions.figureNumber:` If specified, this will set the figure number of the produced figure. Otherwise the figure number will be randomly generated (and probably large).

`localOptions.fileName:` The function may save figures according to preferences.

dendrogramConditions

`dendrogramConditions`(RDMs, userOptions[, localOptions])

This function will generate a dendrogram for each RDM in RDMs. The dendrograms will contain condition names and/or dots that are colored according to user's preferences. The input to the module is a dissimilarity matrix and the output is a figure that can be saved in different formats if set by the user (these are set in the userOptions structure). The user can modify detailed settings (e.g. the name under which the graphics will be saved, the figure number, etc.)

RDMs: The RDMs to be operated on.

RDMs is a structure containing RDMs. It can be of whatever dimension but must have fields: RDM, name, color.

userOptions: The options structure.

userOptions.analysisName: A string which is prepended to the saved files.

userOptions.rootPath: A string describing the root path where files will be saved (inside created directories).

userOptions.conditionLabels. Defaults to false.

userOptions.conditionColours: A [nConditions 3]-sized matrix indicating an [R G B] triple colour for each condition.

userOptions.saveFigurePDF: A Boolean value. If true, the figure is saved as a PDF. Defaults to false.

userOptions.saveFigurePS: A Boolean value. If true, the figure is saved as a PS. Defaults to false.

userOptions.saveFigureFig: A Boolean value. If true, the figure is saved as a MATLAB .fig file. Defaults to false.

userOptions.displayFigures: A Boolean value. If true, the figure remains open after it is created. Defaults to true.

localOptions: Further options specific to this function.

localOptions.titleString: If set, this will replace the default title for the dendrogram.

localOptions.alternativeConditionLabels: A cell array containing alternative names for each condition for display on figures. Defaults to be the same as userOptions.conditionLables.

localOptions.useAlternativeConditionLabels: A Boolean value. If true, localOptions.alternativeConditionLabels are used to label the diagram instead of userOptions.conditionLabels.

localOptions.figureNumber: If specified AND if only one figure will be produced, this will set the figure number of the produced figure. Otherwise the figure number will be randomly generated (and probably large).

Figures generate by this function may be saved according to preferences.

`MDSConditions.m`

`MDSConditions(RDMs, userOptions[, localOptions])`

This function performs first-order MDS, visualising the dissimilarities between brain response patterns. If specified in userOptions, a coloured dot will be drawn for each condition and a text label is displayed for each condition as well. The user can omit the text labels by defining them as empty strings. The function will generate a separate MDS plot for each RDM in the argument `RDMs`.

RDMs: A structure of RDMs. All RDMs in here will be concatenated and have their condition MDS plots displayed.

userOptions: The options structure.

userOptions.analysisName: A string which is prepended to the saved files.

userOptions.rootPath: A string describing the root path where files will be saved (inside created directories).

userOptions.saveFigurePDF: A Boolean value. If true, the figure is saved as a PDF. Defaults to false.

userOptions.saveFigurePS: A Boolean value. If true, the figure is saved as a PS. Defaults to false.

userOptions.saveFigureFig: A Boolean value. If true, the figure is saved as a MATLAB .fig file. Defaults to false.

userOptions.displayFigures: A Boolean value. If true, the figure remains open after it is created. Defaults to true.

userOptions.conditionLabels: A cell array containing the names of the conditions in this experiment.

userOptions.criterion: The criterion which will be minimised to optimise the MDS arrangement. Defaults to metric stress.

userOptions.rubberbands: Boolean value. If true, rubberbands indicating MDS distortion are drawn on the MDS plot. Defaults to true.

userOptions.conditionColours: a [nConditions 3]-sized matrix indicating an [R G B] triple colour for each condition. Defaults to all black.

userOptions.convexHulls: a vector of length equal to the number of conditions. Each entry in the vector corresponds to the same-index condition, and the number for this entry represents a category for this condition. Convex hulls are drawn around all conditions of the same category on the MDS plots, coloured by the first colour in userOptions.conditionColours for the points in this category. For example: [1 1 1 1 2 2 2 2 3 3 3 3 4 4 4 4] would represent four categories, each with four conditions. If unset, convex hulls will not be drawn.

localOptions: Further options specific to this function.

localOptions.titleString: If set, this will replace the default title for the dendrogram.

localOptions.alternativeConditionLabels: A cell array containing alternative names for each condition for display on figures.

localOptions.figureNumber: If specified AND if only one figure will be produced, this will set the figure number of the produced figure. Otherwise the figure number will be randomly generated (and probably large).

pairwiseCorrelateRDMs

parwiseCorrelateRDMs({RDMs, [RDMs2, ...,]}, userOptions[, localOptions])

This function will compute and display a second-order similarity matrix from RDMs.

RDMs, RDMs2, ...: Structures of RDMs. All RDMs in here will be concatenated and pairwise correlated to create a large second-order similarity matrix.

userOptions: The options structure.

userOptions.analysisName: A string which is prepended to the saved files.

userOptions.rootPath: A string describing the root path where files will be saved (inside created directories).

userOptions.distanceMeasure: A string descriptive of the distance measure to be used to compare two RDMs. Defaults to 'Spearman' but any Matlab distance measure may be used.

userOptions.saveFigurePDF: A Boolean value. If true, the figure is saved as a PDF. Defaults to false.

userOptions.saveFigurePS: A Boolean value. If true, the figure is saved as a PS. Defaults to false.

userOptions.saveFigureFig: A Boolean value. If true, the figure is saved as a MATLAB .fig file. Defaults to false.

userOptions.displayFigures: A Boolean value. If true, the figure remains open after it is created. Defaults to true.

userOptions.colourScheme: A colour scheme for the RDMs. Defaults to jet(64).

localOptions: Further options specific to this function.

localOptions.fileName: Whatever is in this string will replace the '%' in the saved filename 'analysisName_%secondOrderSM[.pdf]' under which figures may be saved. Defaults to empty.

localOptions.figureNumber: If specified, this will set the figure number of the produced figure. Otherwise the figure number will be randomly generated (and probably large).

This function may save the displayed figures according to preferences.

MDSRDMs

`MDSRDMs({RDMs,[RDMs2,...]}, userOptions, localOptions)`

This function performs second-order MDS, visualising the dissimilarities between RDMs. A point in a second-order MDS arrangement represents an RDM. The arrangement reflects the extent to which regional representational geometries are similar.

RDMs, RDMs2, ...: Structure of RDMs. All RDMs in here will be placed on an MDS plot.

userOptions: The options structure.

userOptions.analysisName: A string which is prepended to the saved files.

userOptions.rootPath: A string describing the root path where files will be saved (inside created directories).

userOptions.saveFigurePDF: A Boolean value. If true, the figure is saved as a PDF. Defaults to false.

userOptions.saveFigurePS: A Boolean value. If true, the figure is saved as a PS. Defaults to false.

userOptions.saveFigureFig: A Boolean value. If true, the figure is saved as a MATLAB .fig file. Defaults to false.

userOptions.displayFigures: A Boolean value. If true, the figure remains open after it is created. Defaults to true.

userOptions.criterion: The criterion which will be minimised to optimise the MDS arrangement. Defaults to metric stress.

userOptions.rubberbands: Boolean value. If true, rubberbands indicating MDS distortion are drawn on the MDS plot. Defaults to true.

localOptions: Further options specific to this function.

localOptions.titleString: If set, this will replace the default title for the MDS plots.

localOptions.name

localOptions.figureNumber: If specified, this will set the figure number of the produced figure. Otherwise the figure number will be randomly generated (and probably large).

compareRefRDM2candRDMs (key function for statistical inference)

The key function for statistical inference in the toolbox is compareRefRDM2candRDMs.m. This function implements all inference procedures described in the paper. Here we describe these procedures in greater detail. We first explain the general functionality, with some redundancy to the main paper. We then define the usage and all inputs and outputs of the function in detail. The text below is identical to the help text of compareRefRDM2candRDMs.m, but Figure 2 (identical to Fig. S1 in the supplement of Nili et al. and reproduced here for convenience) has been added to give an overview of the statistical inference methods, the circumstances under which each is available, and the default choices.

GENERAL PURPOSE

The function compareRefRDM2candRDMs.m compares a reference RDM to multiple candidate RDMs. For example, the reference RDM could be a brain region's RDM and the candidate RDMs could be multiple computational models. Alternatively, the reference RDM could be a model RDM and the candidate RDMs could be multiple brain regions' RDMs. More generally, the candidate RDMs could include both model RDMs

and RDMs from brain regions, and the reference RDM could be either a brain RDM or a model RDM. In all these cases, one reference RDM is compared to multiple candidates.

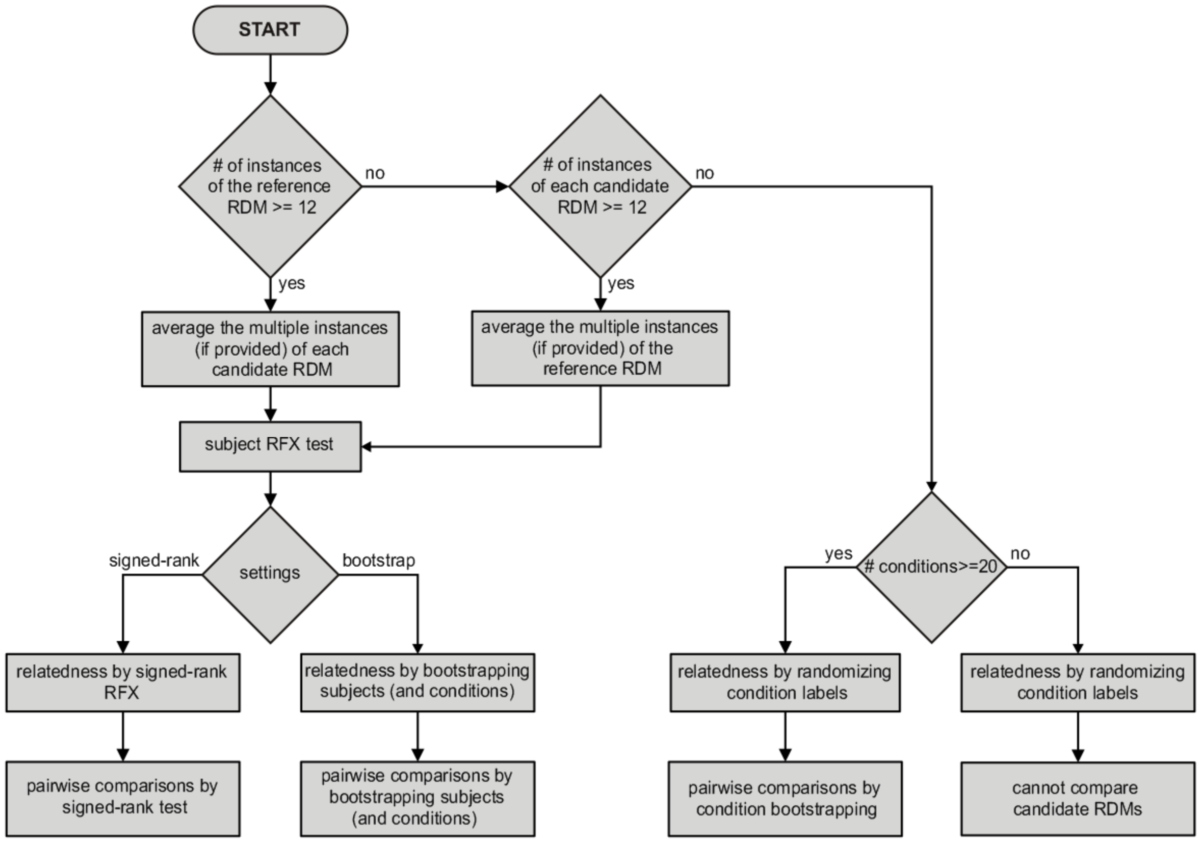

Figure 2: Decision process for selection of statistical tests. (This figure is identical with Fig. S1 in the supplement of Nili et al. and reproduced here for convenience.) The flowchart above shows the default decision process by which the statistical inference procedures are chosen in the toolbox. The analyses in Figures 4 and 5 of the paper correspond to paths in the flowchart that lead to the leftmost (simulation in Figure 4) and second from right (real data in Figure 5) box at the bottom. Note that the flowchart does not capture all possibilities. For example, the fixed-effects condition-label randomization test of RDM relatedness can be explicitly requested, even when there are 12 or more subjects' estimates of the reference RDM and the random-effects signed-rank test would be chosen by default.

TESTING RDM CORRELATIONS

The function compares the reference RDM to each of the candidates, tests each candidate for significant RDM correlation (test dependent on input data and `userOptions`) and presents a bar graph of the RDM correlations in descending order from left to right (best candidates on the left) by default, or in the order in which the candidate RDMs are passed. In addition, pairwise comparisons between the candidate RDMs are performed to assess, for each pair of candidate RDMs, which of the two RDMs is more consistent with the reference RDM. A significant pairwise difference is indicated by a horizontal line above the corresponding two bars. Each bar comes with an error bar, which indicates the standard error, estimated by the same procedure as is used for the pairwise candidate comparisons (dependent on input data and `userOptions`, see below).

Statistical inference on the correlation between the reference RDM and each candidate RDM is performed using a one-sided Wilcoxon signed-rank across subjects by default. When the number of subjects is insufficient for this test to be valid (<12), or when requested in `userOptions`, the test is instead performed by condition-label randomisation. By default, the false-discovery rate is controlled for these tests across candidate models. When requested in `userOptions`, the familywise error rate is controlled instead.

COMPARISONS BETWEEN CANDIDATE RDMS

For the comparisons between candidate RDMs as well, the inference procedure depends on the input data provided and on `userOptions`. By default, a signed-rank test across repeated measurements of the RDMs (e.g. multiple subjects or sessions) is performed. Alternatively, these tests are performed by bootstrapping of the subjects and/or conditions set. Across the multiple pairwise comparisons, the function controls the familywise error rate (Bonferroni method) or the false-discovery rate (Benjamini and Hochberg, 1995).

CEILING UPPER AND LOWER BOUNDS

If multiple instances of the reference RDM are passed (typically representing estimates for multiple subjects), a ceiling estimate is indicated by a gray transparent horizontal bar. The ceiling is the expected value, given the noise (i.e. the variability across subjects), of the average correlation of the true model's RDM with the single-subject RDMs. The upper and lower edges of the ceiling bar are upper- and lower-bound estimates for the unknown true ceiling. The upper bound is estimated by computing the correlation between each subject's RDM and the group-average RDM. The lower bound is estimated similarly, but using a leave-one-out approach, where each subject's RDM is correlated with the average RDM of the other subjects' RDMs. When Pearson correlation is chosen for comparing RDMs (`userOptions.RDMcorrelationType`), the RDMs are first z-transformed. When Spearman correlation is chosen, the RDMs are first rank-transformed. When Kendall's tau-a is chosen, an iterative procedure is used. See main paper for a full motivation for the ceiling estimate and for an explanation of why these are useful upper- and lower-bound estimates. (See also below, under *(5) Estimating the upper bound on the noise ceiling for the RDM correlation*.)

Usage

```
stats_p_r = compareRefRDM2candRDMs(refRDM, candRDMs[, userOptions])
```

ARGUMENTS

`refRDM`

The reference RDM, which can be a square RDM or lower-triangular-vector RDM, or a wrapped RDM (structure specifying a name and colour for colour-coding of the RDM in figures). refRDM may also be a set of independent estimates of the same RDM (square matrices or lower-triangular vectors stacked along the third dimension or a structured array of wrapped RDMs), e.g. an estimate for each of multiple subjects or sessions, which are then used for random-effects inference.

`candRDMs`

A cell array with one cell for each candidate RDM. The candidate RDMs can be square or lower-triangular-vector RDMs or wrapped RDMs. Each candidate RDM may also contain multiple independent estimates of the same RDM, e.g. an estimate for each of multiple subjects or sessions. These can be used for random-effects inference if all candidate RDMs have the same number of independent estimates, greater than or equal to 12. However, if refRDM contains 12 or more independent estimate of the reference RDMs, then random-effects inference is based on these and multiple instances of any candidate RDMs are replaced by their average. In case the dissimilarity for a given pair of conditions is undefined (NaN) in any candidate RDM or in the reference RDM, that pair is set to NaN in all RDMs and treated as a missing value. This ensures that comparisons between candidate RDMs are based on the same set of dissimilarities.

`userOptions.RDMcorrelationType`

The correlation coefficient used to compare RDMs. This is `'Spearman'` by default, because we prefer not to assume a linear relationship between the distances (e.g. when a brain RDM from fMRI is compared to an RDM predicted by a computational model). Alternative definitions are `'Kendall_taua'` (which is appropriate whenever categorical models are tested) and `'Pearson'`. The Pearson correlation coefficient may be justified when RDMs from the same origin (e.g. multiple computational models or multiple brain regions measured with the same method) are compared. For more details on the RDM correlation type, see main paper and Figure S2.

`userOptions.RDMrelatednessTest`

`'subjectRFXsignedRank'` (default): Test the relatedness of the reference RDM to each candidate RDM by computing the correlation for each subject and performing a one-sided Wilcoxon signed-rank test against the null hypothesis of 0 correlation, so as to test for a positive correlation. (Note that multiple independent measurements of the reference or candidate RDMs could also come from repeated measurements within one subject. We refer to the instances as "subjects", because subject random-effects inference is the most common case.)

`'randomisation'`: Test the relatedness of the reference RDM to each candidate RDM by randomising the condition labels of the reference RDM, so as to simulate the null distribution for the RDM correlation with each candidate RDM. In case there are multiple instances of the reference or candidate RDMs, these are first averaged.

`'conditionRFXbootstrap'`: Test the relatedness of the reference RDM to each candidate RDM by bootstrapping the set of conditions (typically: stimuli). For each bootstrap sample of the conditions set, a new instance is generated for the reference RDM and for each of the candidate RDMs. Because bootstrap resampling is resampling with replacement, the same condition can appear multiple times in a sample. This entails 0 entries (from the diagonal of the original RDM) in off-diagonal positions of the RDM for a bootstrap sample. These zeros are treated as missing values and excluded from the dissimilarities, across which the RDM correlations are computed. The p value for a one-sided test of the relatedness of each candidate RDM to the reference RDM is computed as the proportion of bootstrap samples with a zero or negative RDM correlation. This test simulates the variability of the estimates across condition samples and thus supports inference

generalising to the population of conditions (or stimuli) that the condition sample can be considered a random sample of. Note that basic bootstrap tests are known to be slightly optimistic.

`'subjectConditionRFXbootstrap'`: Bootstrap resampling is simultaneously performed across both subjects and conditions. This simulates the greater amount of variability of the estimates expected if the experiment were repeated with a different sample of subjects and conditions. This more conservative test attempts to support inference generalising across both subjects and stimuli (to their respective populations). Again, the usual caveats for basic bootstrap tests apply.

`'none'`: Omit the test of RDM relatedness.

`userOptions.RDMrelatednessThreshold`

The significance threshold (default: 0.05) for testing each candidate RDM for relatedness to the reference RDM. Depending on the choice of multiple testing correction (see next `userOptions` field), this can be the expected false-discovery rate, the familywise error rate, or the uncorrected p threshold.

`userOptions.RDMrelatednessMultipleTesting`

`'FDR'` (default): Control the false-discovery rate across the multiple tests (one for each candidate RDM). With this option, `userOptions.RDMrelatednessThreshold` is interpreted to specify the expected false-discovery rate, i.e. the expected proportion of candidate RDMs falsely declared significant among all candidate RDMs declared significant.

`'FWE'`: Control the familywise error rate. When the condition-label randomisation procedure is selected to test RDM relatedness, then randomisation is used to simulate the distribution of maximum RDM correlations across all candidate RDMs. This method is more powerful than Bonferroni correction when there are dependencies among candidate RDMs. If another test is selected to test RDM relatedness, the Bonferroni method is used. In either case, `userOptions.RDMrelatednessThreshold` is interpreted as the familywise error rate, i.e. the probability of getting any false positives under the omnibus null hypothesis that all candidate RDMs are unrelated to the reference RDM.

`'none'`: Do not correct for multiple testing (not recommended). With this setting, `userOptions.RDMrelatednessThreshold` is interpreted as the uncorrected p threshold.

`userOptions.candRDMdifferencesTest`

`'subjectRFXsignedRank'` (default, data permitting): For each pair of candidate RDMs, perform a statistical comparison to determine which candidate RDM better explains the reference RDM by using the variability across subjects of the reference or candidate RDMs. The test is a two-sided Wilcoxon signed-rank test of the null hypothesis that the two RDM correlations (refRDM to each of the candidate RDMs) are equal. This is the default test when multiple instances of the reference RDM (typically corresponding to multiple subjects) or a consistent number of multiple instances of each candidate RDMs is provided and the number of multiple instances is 12 or greater. This test supports inference generalising to the population of subjects (or repeated measurements) that the sample can be considered a random sample of.

'subjectRFXbootstrap': For each pair of candidate RDMs, perform a two-sided statistical comparison to determine which candidate RDM better explains the reference RDM by bootstrapping the set of subjects. For each bootstrap sample of the subjects set, the RDMs are averaged across the bootstrap sample and the difference between the two RDM correlations (refRDM to each of the candidate RDMs) is computed. The p value is estimated as the proportion of bootstrap samples further in the tails (symmetrically defined for a two-sided test) than 0. This test simulates the variability of the estimates across subject samples and thus supports inference generalising to the population of subjects (or repeated measurements) that the sample can be considered a random sample of. The usual caveats for basic bootstrap tests apply.

'conditionRFXbootstrap': For each pair of candidate RDMs, perform a two-sided statistical comparison to determine which candidate RDM better explains the reference RDM by bootstrapping the set of conditions (typically: stimuli). For each bootstrap sample of the conditions set, a new instance is generated for the reference RDM and for each of the candidate RDMs. Because bootstrap resampling is resampling with replacement, the same condition can appear multiple times in a sample. This entails 0 entries (from the diagonal of the original RDM) in off-diagonal positions of the RDM for a bootstrap sample. These zeros are treated as missing values and excluded from the dissimilarities, across which the RDM correlations are computed. The p value for the two-sided test of the difference for each pair of candidate RDMs is computed as for the setting subjectRFXbootstrap (see above). This test simulates the variability of the estimates across condition samples and thus supports inference generalising to the population of conditions (typically stimuli) that the condition sample can be considered a random sample of. Again, the usual caveats for bootstrap tests apply.

'subjectConditionRFXbootstrap': Bootstrap resampling is simultaneously performed across both subjects and conditions. This simulates the greater amount of variability of the estimates expected if the experiment were repeated with a different sample of subjects and conditions. This more conservative test attempts to support inference generalising across both subjects and stimuli (to their respective populations. However, the usual caveats for bootstrap tests apply.

'none': Omit the pairwise tests of candidate RDMs comparing their relative ability to explain the reference RDM.

userOptions.candRDMdifferencesThreshold

The significance threshold for comparing each pair of candidate RDMs in terms of their relatedness to the reference RDM. Depending on the choice of multiple testing correction (see next userOptions field), this can be the expected false-discovery rate, the familywise error rate, or the uncorrected p threshold.

userOptions.candRDMdifferencesMultipleTesting

'FDR': Control the false-discovery rate across the multiple tests (one for each candidate RDM). With this option, userOptions.candRDMdifferencesThreshold is interpreted to specify the expected false-discovery rate, i.e. the expected proportion of pairs of candidate RDMs falsely declared significantly different among all pairs of candidate RDMs declared significantly different (in their relatedness to the reference RDM).

'FWE': Control the familywise error rate. With this option, the Bonferroni method is used to ensure that the familywise error rate is controlled. userOptions.candRDMdifferencesThreshold is interpreted as the familywise error rate, i.e. the probability of getting any false positives under the omnibus null hypothesis that all pairs of candidate RDMs are equally related to the reference RDM.

'none': Do not correct for multiple testing (not recommended). userOptions. candRDMdifferencesThreshold is interpreted as the uncorrected p threshold.

userOptions.nRandomisations

The number of condition-label randomisations (default: 10,000) used to simulate the null distribution that the reference RDM is unrelated to each of the candidate RDMs.

userOptions.nBootstrap

The number of bootstrap resamplings (default: 1,000) used in all selected bootstrap procedures (relatedness test, candidate comparison tests, error bars).

userOptions.plotpValues

This option controls how the significance of the RDM relatedness tests is indicated. If set to '*' (default), then an asterisk is plotted on the bar for each candidate RDM that is significantly related to the reference RDM. Significance depends on the test (see above) and on userOptions. RDMrelatednessThreshold (default: 0.05) and on userOptions. RDMrelatednessMultipleTesting (default: 'FDR'). Asterisks mark candidate RDMs that are significant at the specified threshold and with the chosen method for accounting for multiple testing. If set to '=', then the uncorrected p value is plotted below the bar for each candidate RDM, and it is plotted in bold type if it is significant by the criteria explained above.

userOptions.barsOrderedByRDMCorr

This option controls the order of the displayed bars (default: true). If set to true, bars corresponding to candidate RDMs are displayed in descending order (from left to right) according to their correlation to the reference RDM. Otherwise, bars are displayed in the order in which the candidate RDMs are passed.

userOptions.figureIndex

This option enables the user to specify the figure numbers for the two created figures (default: [1 2]). The first figure contains the bar graph and the second contains matrices indicating the significance of the pairwise candidate RDM comparisons. The first panel shows the uncorrected p matrix. The second panel shows the thresholded uncorrected p matrix. The third panel shows the FDR-thresholded p matrix. The fourth panel shows the Bonferroni-thresholded p matrix.

userOptions.resultsPath

This argument specifies the absolute path in which both figures are to be saved (default: pwd, i.e. current working directory).

userOptions.saveFigurePDF

If set to true (default), the figures are saved in PDF format in userOptions.resultsPath.

userOptions.saveFigurePS

If true (default: false), the figures are saved in post-script format in userOptions.resultsPath.

userOptions.saveFigureFig

If true (default: false), the figures are saved in Matlab .fig format in userOptions.resultsPath.

userOptions.figure1filename

The filename for the bargraph figure, if chosen to be saved (default: 'compareRefRDM2candRDMs_barGraph').

userOptions.figure2filename

The filename for the p-value display figure, if chosen to be saved (default: 'compareRefRDM2candRDMs_RDMcomparisonPvalues').

Return values

stats_p_r

Structure containing numerical statistical results, including effect sizes and p values.

stats_p_r.candRelatedness_r: Average correlations to reference RDM.

stats_p_r.candRelatedness_p: Corresponding uncorrected p values.

stats_p_r.SEs: Standard errors of average RDM correlations.

stats_p_r.candDifferences_r: Matrix of bar-height differences (i.e. average RDM-correlation differences).

stats_p_r.candDifferences_p: Matrix of p values for all pairwise candidate comparisons.

stats_p_r.orderedCandidateRDMnames: Candidate RDM names in the order in which the bars are displayed (also the order used for the return values).

stats_p_r.ceiling: Ceiling lower and upper bounds.

TOOLBOX ENGINES

Engines are self-contained Matlab scripts, each of which serves a particular purpose. This section contains the help text of all 76 engines of the RSA toolbox.

defineUserOptions

`defineUserOptions` is a function to be edited by the user, which defines the structure `userOptions` (see below) containing the preferences and details for a particular project. For example, it defines where the data is stored, what colours should be used consistently across analyses to code for ROIs, models, and conditions, and whether resulting figures are to be saved, and if so in which format (PS, PDF, JPG, fig). It is helpful to define these variables once for each project, so as to use consistent conventions across all analyses.

userOptions --- The options structure.

`userOptions.analysisName`: A string which is prepended to the saved files. This means that files from the same project will tend to be grouped together.

`userOptions.rootPath`: A string describing the root path where files will be saved. If the path does not exist, it will be created.

`userOptions.maskPath`: A string describing the path to the location of the files for the definition of RoI masks.

`userOptions.subjectNames`: A cell array containing strings identifying the subject names. Defaults to the field names in fullBrainVols (the output of `fMRIDataPreparation, see later).`

`userOptions.maskNames`: A cell array containing strings identifying the mask names. Defaults to the field names of the first subject of binaryMasks_nS.

`userOptions.betaPath`: A string which contains the path to the location of the beta images. It can contain the following wildcards which would be replaced as indicated:

[[`subjectName`]]: To be replaced with the name of each subject where appropriate.

[[`betaIdentifier`]]: To be replaced by filenames as provided by betaCorrespondence.

`userOptions.conditionLabels`: A cell array containing the names of the conditions in the experiment.

`userOptions.voxelSize`: A triple consisting of the [x y z] dimensions of each voxel in mm.

`userOptions.structuralsPath`: A string which contains the path to the location of the structural images and the normalisation warp definition file. It can contain the following wildcards which would be replaced as indicated:

[[`subjectName`]]: To be replaced with the name of each subject where appropriate.

userOptions.distanceMeasure: A string descriptive of the distance measure to be used to compare two RDMs. Defaults to 'Spearman'. Other options include any standard Matlab correlation type (also see rankCorr_Kendall_tau_a.m)

userOptions.saveFigurePDF: A boolean value. If true, the figure is saved as a PDF (Defaults to true.)

userOptions.saveFigurePS: A boolean value. If true, the figure is saved as a PS. (Defaults to false.)

userOptions.saveFigureFig: A boolean value. If true, the figure is saved as a MATLAB .fig file. (Defaults to false.)

userOptions.saveFiguresJpg: A boolean value. If true, the figure is saved as a jpeg image.

userOptions.displayFigures: A boolean value. If true, the figure remains open after it is created. (Defaults to true.)

userOptions.imagelables: Defaults to empty (no image labels). Future releases of the toolbox will allow the user to display image labels besides the RDMs.

userOptions.rankTransform: Boolean value. If true, values in each RDM are separately transformed to lie uniformly in [0,1] with ranks preserved. If false, true values are represented. (Defaults to true.) This is for displaying RDMs only.

userOptions.criterion: The criterion which will be minimised to optimise the MDS arrangement. (Defaults to metric stress.)

userOptions.rubberbands: Boolean value. If true, rubberbands indicating MDS distortion are drawn on the MDS plot. (Defaults to true.)

userOptions.distance: A string indicating the distance measure with which to calculate the RDMs. (Defaults to 'Correlation'.)

userOptions.RoIColor: A triple indicating the [R G B] value of the colour which should be used to indicated data RDMs on various diagrams. (Defaults to black: [0 0 0].)

userOptions.ModelColor: A triple indicating the [R G B] value of the colour which should be used to indicated model RDMs on various diagrams. (Defaults to black: [0 0 0].)

userOptions.nResamplings: How many bootstrap resamplings should be performed? (Defaults to 1000.)

userOptions.resampleSubjects: Boolean. If true, subjects will be bootstrap resampled. (Defaults to false.)

userOptions.resampleConditions: Boolean. If true, conditions will be bootstrap resampled. (Defaults to true.)

userOptions.dotSize: A number specifying the size of the circles used to indicate points in MDS plots. (Defaults to 8.)

userOptions.significanceTestPermutations: An integer which describes the number of random permutations to be used to calculate significance (Defaults to 10,000.)

userOptions.conditionColours: A [nConditions 3]-sized matrix indicating an [R G B] triple colour for each condition. (Defaults to all black.)

userOptions.convexHulls: A vector of length equal to the number of conditions. Each entry in the vector corresponds to the same-index condition, and the number for this entry represents a category for this condition. Convex hulls are drawn around all conditions of the same category on the MDS plots, coloured by the first colour in userOptions.conditionColours for the points in this category. For example: [1 1 1 1 2 2 2 2 3 3 3 3 4 4 4 4] would represent four categories, each with four conditions. If unset, convex hulls will not be drawn.

userOptions.colourScheme: A colour scheme for the RDMs. This field defaults to the colormap given by the RDMcolormap function (see later).

setIfUnset

`options=setIfUnset(options,field,value)`

If `options.(field)` is empty or doesn't exist, this function sets `options.(field)` to `value`.
This engine gets as its input a structure array and sets a field where the field does not exist. In case the field already exists, the value is replaced by the third argument of the function, `value`.

vectorizeRDM

`RDM=vectorizeRDM(RDM)`

Converts `RDM` to lower-triangular form (row vector) (or leaves it in that form). In cases where the input (`RDM`) is a vector, the output would be the same as the input (row or column vector). If the input is a 2D matrix (e.g. an RDM) the output would be a (row) vector of the lower-triangular entries. This function does not accept a wrapped RDM as its input. Note that if the input matrix is non-symmetric, vectorizeRDM only outputs the lower triangular entries as a row vector.

unwrapRDMs

`[RDMs,nRDMs]=unwrapRDMs(RDMs_struct)`

Unwraps dissimilarity matrices of a structured array (wrapped RDMs) with metadata (`RDMs_struct`) by extracting the dissimilarity matrices (in square or lower triangle form) and lining them up along the third dimension. (if they are already in that format they are handed back unchanged.) It must be noted that different dissimilarity sets (i.e. lower-triangular or squareform RDMs) must be different fields of the input structure. The only requirement is that the dissimilarities should be stored in a field called `'RDM'`. `nRDMs` is the number of wrapped RDMs in the input. in case where all the input RDMs are in lower-triangular or square form, the output would be in the same format as well (RDMs will be stacked along a third dimension). If the input RDMs are of mixed type, they are all made square. Also, if RDMs in the input are of different sizes, they'll all be returned as squares with NaNs outside padding them to the size of the largest.

wrapRDMs

`RDMs_struct=wrapRDMs(RDMs,refRDMs_struct)`

Wraps similarity matrices `RDMs` (in square or upper triangle form) into a structured array with metadata copied from `refRDMs_struct` (which needs to have the same number of RDMs). These structured arrays are used to pair RDMs up with metadata so that they may be appropriately coloured or labelled in figures. If the input RDMs are already in a wrapped form, then the wrapping (metadata) is replaced by that of `refRDMs_struct`.

In cases where the user wants to define names or specify colours for the dissimilarity vector or matrices they need to specify this in the `refRDMs_struct using 'name' and 'colour' fields respectively`. If none are specified, generic names and values are selected and returned in the `'name'` and `'color'` fields.

averageRDMs_subjectSession

`RDMs = averageRDMs_subjectSession(RDMs)`: returns the input unchanged (no operation).

`RDMs = averageRDMs_subjectSession(RDMs, 'subject')`: computes the subject-averaged RDMs.

`RDMs = averageRDMs_subjectSession(RDMs, 'session')`: computes the session-averaged RDMs.

`RDMs = averageRDMs_subjectSession(RDMs, 'subject', 'session')`: first averages RDMs across subjects and then sessions.

`RDMs = averageRDMs_subjectSession(RDMs, 'session', 'subject')`: first averages the RDMs across sessions and then subjects.

Averages are computed from a structure of RDMs which is assumed to be in a 3-dimensional array (or structured array) of size [nMasks, nSubjects, nSessions]. A structure is returned containing RDMs averaged along the specified dimension based on the arguments.

The first dimension is assumed to represent the number of RoIs used in the analysis, the second the number of subjects, and the third the number of scanning sessions, with one RDM per RoI, subject and session. If RDMs given to this function should be kept in this format so that the averaging operates as expected.

The naming convention is the following:

`RDMs(1).name = 'RoiName | subjectName | sessionName/number';`

If this convention is followed for the input structure of RDMs, the same convention will be followed for the output structure, with the subject/session segments of the name removed based on which averaging dimensions were specified.

Brightness

`b=brightness(RGBrows)`

Given triples of RGB values, this function returns the overall brightness vector. This is performed on each row of the input (RGBrows) independently.

fMRIDataPreparation

[fullBrainVols =] fMRIDataPreparation(betaCorrespondence, userOptions)

fMRIDataPreparation is a function designed to take fMRI data and load it into the correct format for the rest of the toolbox to use.

betaCorrespondence: The array of beta filenames.

betas(condition, session).identifier is a string which refers to the filename (not including path) of the SPM beta image. Alternatively, this can be the string 'SPM', in which case the SPM metadata will be used to infer this information, provided that userOptions.conditionLabels is set, and the condition labels are the same as those used in SPM.

userOptions: The standard options structure containing the following fields:

userOptions.analysisName: A string which is prepended to the saved files. userOptions.rootPath: A string describing the root path where files will be saved (inside created directories).

userOptions.subjectNames: A cell array containing strings identifying the subject names. Defaults to the field names in fullBrainVols.

userOptions.betaPath: A string which contains the absolute path to the location of the beta images. It can contain the following wildcards which would be replaced as indicated:

"[[subjectName]]": To be replaced with the name of each subject where appropriate.

"[[betaIdentifier]]": To be replaced by filenames as provided by betaCorrespondence.

userOptions.conditionLabels: A Cell array containing the names of the conditions in this experiment. This must be set if the "SPM" option is used.

The following files are saved by this function:

userOptions.rootPath/ImageData/

userOptions.analysisName_ImageData.mat contains the raw beta images in a structure such that fullBrainVols.(subject) is a [nVoxels nConditions nSessions] matrix.

userOptions.rootPath/Details/
userOptions.analysisName_fMRIDataPreparation_Details.mat contains the userOptions for this execution of the function and a timestamp.

colorScale

`cols=colorScale(anchorCols,nCols,monitor)`
Linearly interpolates between a set of given 'anchor' colours (`anchorCols`) to give `nCols` (number of colours) and displays them if monitor is set.

rankTransform

`rankArray=rankTransform(array,scale01)`

This function transforms the array `'array'` by replacing each element by its rank in the distribution of all its elements. If `scale01` is set to true, the output values are further linearly scaled to lie between 0 and 1, after rank transforming all the non-`NaN` entries. If equal-value entries are present in array, they are randomly assigned adjacent ranks.

rankTransform_equalsStaysEqual

`rankMat=rankTransform_equalsStayEqual(mat,scale01)`

The function transforms the matrix `mat` by replacing each element by its rank in the distribution of all its elements. If scale01 is set to true the ranked elements are linearly scaled to lie between 0 and 1. As indicated by its name (and unlike the function rankTransform), equal values in mat are assigned equal ranks by assigning the mean scaled rank to all the equal entries.

`NaNs` are ignored in this process.

FDRthreshold

`pThreshold=FDRthreshold(pMap,q,binaryBrainMask,assumePositiveDependence)`

This function is used to determine the p value, at which the 3D map of p values `pMap` needs to be thresholded in order for the average false discovery rate (FDR) to be below `q`.

`pMap` the 3D map of p values

[`q`] the average false discovery rate the researcher is willing to accept. (the actual false discovery rate can be larger, but its average is guaranteed to be smaller than q.)

this argument is optional and defaults to 0.05.

[`binaryBrainMask`] a binary 3D map whose size must match `pMap`. Only voxels marked by a nonzero entry in `binaryBrainMask` are considered in determining the threshold. Specifying this mask is highly recommended, because it reduces the expected number of falsely positive voxels under the null hypothesis for any given threshold and increases the sensitivity with which functional regions are detected. However, this argument is optional. If it is omitted the whole volume will be considered.

[assumePositiveDependence] optional argument specifying whether no assumptions at all or very weak assumptions are to be made about the joint distribution of the p values. If assumePositiveDependence is 1 (default), it is assumed that the p-values are either INDEPENDENT or POSITIVELY DEPENDENT (Benjamini and Yekutieli, 2001), i.e. the noise in the data is gaussian with nonnegative correlation across voxels. This assumption is usually reasonable for fMRI data. It also appears reasonable for p values stemming from local-pattern-effect mapping. If assumePositiveDependence is 0, no assumptions are made about the joint distribution of the p values. This comes at a cost in sensitivity, i.e. the resulting thresholds will be smaller, marking fewer voxels. By default, assumePositiveDependence is 1 and the weak assumptions (as described) are made.

This function returns the following value:

pThreshold the critical p value, at which pMap is to be thresholded in order to ensure that the average false discovery rate does not exceed q.

RDMcolormap

`cols=RDMcolormap`

This function provides a convenient colormap for visualizing dissimilarity matrices. It goes from blue to yellow and has grey for intermediate values.

Deunderscore

`stringORstringInCell=deunderscore(stringORstringInCell)`

Gets an input string and replaces the underscores ('_') with hyphens ('-').This avoids the problems with displaying the names in which the first character after the underscore would be displayed as an subscript index. The input can either be a string or a cell array of strings.

setPaperToFigPos

`F = setPapertoFigPos(F)`

This function changes the paper size of a figure F to match the space the figure takes up on the page. This is mainly since it allows user to set the 'position' argument to the desired size and then have the resulting PS/EPS/PDFs come out without a bunch of whitespace. The

showRDMs

```
showRDMs(RDMs,figI,rankTransform01,clims,showColorbar, aspect, imagelabels, colourScheme)
```

Visualizes one or more RDMs. RDMs is a structure of RDMs (wrapped RDMs). Only the 'RDM' and 'name' fields are required for this function. figI is the figure number in which the RDMs are displayed. If rankTransform01 is defined, the dissimilarities are rankTransformed before visualization. clims is a 1x2

vector specfying the lower and upper limits for displayed dissimilarities. If `showColorbar` is set, the colorbar legend would also be displayed in a separate panel. `aspect is a float representing the ratio` numbers of horizontal panels to vertical panels, though it is only treated as a guide to the function. `colourScheme is` the colour scheme for displaying dissimilarities (defined by `RDMcolormap` by default). `imagelabels is` a structure containing the image maps for images that would be displayed on an RDM.
It must be noted that except from RDMs, all other arguments are optional.

addImageSequenceToAxes

`addImageSequenceToAxes(ax,imagelabels)`

This function places a set of images at equal distances along a line in a 2d coordinate system, which is then mapped to the axes of the RDM in `ax`.
`imagelabels` is a struct with the following fields:

- `images` : struct containing
- `image` : RGB image matrix (x,y,3)
- `alpha`: optional alpha layer for image
- `sequence` : vector of custom indices for images. Optional.
- `nRows` : number of rows/columns to plot labels in. Default 2.
- `transparentCol` : Remove background based on rgb colour. Default undefined.
- `blackdisks` : Logical. Place black disks under images. Default value is false.

betaCorrespondence

`betas = betaCorrespondence();`

This is a simple function which should combine three things:

`preBeta`: a string which is at the start of each file containing a beta image, `betas`: a struct indexed by (session,condition) containing a sting unique to each beta image and `postBeta`: a string which is at the end of each file containing a beta image, not containing the file .suffix
use "[[`subjectName`]]" as a placeholder for the subject's name as found in `userOptions.subjectNames` if necessary For example, in an experiment where the data from subject1 (subject1 name) is saved in the format:
`subject1Name_session1_condition1_experiment1.img` and similarly for the other conditions, one could use this function to define a general mapping from experimental conditions to the path where the brain responses are stored. If the paths are defined for a general subject, the term [[`subjectName`]] would be iteratively replaced by the subject names as defined by `userOptions.subjectNames`.

Note that this function could be replaced by an explicit mapping from experimental conditions and sessions to data paths.

handleCurrentFigure

`handleCurrentFigure(fileName, userOptions)`

This is a function which will, based on the preferences set in `userOptions`, save the current figure as a .pdf, a .ps or a .fig; either leaving it open or closing it.

`fileName` --- The name of the file to be saved.
`userOptions` --- The options struct.
`userOptions.analysisName`: A string which is prepended to the saved files.
`userOptions.rootPath`: A string describing the root path where files will be saved (inside created directories).
`userOptions.saveFiguresPDF`: A Boolean value. If true, the figure is saved as a PDF (defaults to false).
`userOptions.saveFiguresPS`: A Boolean value. If true, the figure is saved as a PS (defaults to false).
`userOptions.saveFiguresFig`: A Boolean value. If true, the figure is saved as a MATLAB .fig file (defaults to false).
`userOptions.saveFiguresEps`: A Boolean value. If true, the figure is saved as an EPS (defaults to false).

`userOptions.saveFiguresJpg`: A Boolean value. If true, the figure is saved as a jpeg image.
`userOptions.displayFigures`: A Boolean value. If true, the figure remains open after it is created (defaults to true).

exportCurrentFigAsPostscript

`exportCurrentFigAsPostscript(filespec,appendFlag,userOptions)`

This function exports the current figures to the file [`filespec`,'.ps'] in postscript format. if `appendFlag` is 0 any existing file [`filespec`,'.ps'] is overwritten. If `appendFlag` is 1 the figure is appended to the existing postscript file [`filespec`,'.ps']. if `appendFlag` is 3 (default) the figure is appended to the existing postscript file [`filespec`,'.ps'] and exported to a separate file `[filespec,'_',num2str(gcf),'.ps'].`

exportCurrentFigAsPDF

`exportCurrentFigAsPDF(filespec,userOptions)`

This function exports the current figures to the file [filespec,'.pdf'] in pdf format. `filespec` is the full path (including the file name) of the file to be saved.
`userOptions` can be used to modify the dots per inch (dpi) or the tightness of the figure in the printed page. This can be done by defining `userOptions.dpi` or setting the `userOptions.tightInset` respectively.

Exportfig

`exportfig(H, FILENAME)`

This function exports a figure to Encapsulated Postscript.

`exportfig` writes the figure `H` to `FILENAME`. H is a figure handle and `FILENAME` is a string that specifies the name of the output file.

exportfig(...,PARAM1,VAL1,PARAM2,VAL2,...) specifies parameters that control various characteristics of the output file.

Format Paramter:

'Format' one of the strings 'eps','eps2','jpeg','png','preview' specifies the output format. Defaults to 'eps'.

The output format 'preview' does not generate an output file but instead creates a new figure window with a

preview of the exported figure. In this case the FILENAME parameter is ignored.

'Preview' one of the strings 'none', 'tiff' specifies a preview for EPS files. Defaults to 'none'.

Size Parameters:

'Width' a positive scalar specifies the width in the figure's PaperUnits

'Height' a positive scalar specifies the height in the figure's PaperUnits

Specifying only one dimension sets the other dimension so that the exported aspect ratio is the same as the

figure's current aspect ratio. If neither dimension is specified the size defaults to the width and height from the figure's PaperPosition.

Rendering Parameters:

'Color' one of the strings 'bw', 'gray', 'cmyk'

'bw' specifies that lines and text are exported in black and all other objects in grayscale

'gray' specifies that all objects are exported in grayscale

'cmyk' specifies that all objects are exported in color

using the CMYK color space

'Renderer' one of the strings 'painters', 'zbuffer', 'opengl' specifies the renderer to use
'Resolution' a positive scalar specifies the resolution in dots-per-inch.

The default color setting is 'bw'.

Font Parameters:

'FontMode' one of the strings 'scaled', 'fixed'

'FontSize' a positive scalar

in 'scaled' mode multiplies with the font size of each

text object to obtain the exported font size

in 'fixed' mode specifies the font size of all text objects in points

'FontEncoding' one of the strings 'latin1', 'adobe' specifies the character encoding of the font

If FontMode is 'scaled' but FontSize is not specified then a scaling factor is computed from the ratio of the size of the exported figure to the size of the actual figure. The minimum font size allowed after scaling is 5 points.

If FontMode is 'fixed' but FontSize is not specified then the exported font sizes of all text objects is 7 points.

The default 'FontMode' setting is 'scaled'. Line Width Parameters:

'LineMode' one of the strings 'scaled', 'fixed'

'LineWidth' a positive scalar the semantics of LineMode and LineWidth are exactly the

same as FontMode and FontSize, except that they apply to line widths instead of font sizes. The minumum line

width allowed after scaling is 0.5 points. If LineMode is 'fixed' but LineWidth is not specified

then the exported line width of all line objects is 1 point.

Examples:

exportfig(gcf,'fig1.eps','height',3); Exports the current figure to the file named 'fig1.eps' with

a height of 3 inches (assuming the figure's PaperUnits is inches) and an aspect ratio the same as the figure's aspect ratio on screen.

```
exportfig(gcf, 'fig2.eps', 'FontMode', 'fixed',...

    'FontSize', 10, 'color', 'cmyk' );
```

Exports the current figure to 'fig2.eps' in color with all text in 10 point fonts. The size of the exported figure is

the figure's PaperPostion width and height.

concatRDMs_unwrapped

RDMs=concatRDMs_unwrapped(varargin)

Concatenates dissimilarity sets (RDMs in either square or lower-triangular form). All inputs should have the same number of dissimilarity entries.

concatRDMs

RDMs=concatRDMs(varargin)

Concatenates representational dissimilarity matrices and returns the concatenated RDMs in a wrapped structure.

rankCorr_Kendall_taua

`taua=rankCorr_Kendall_taua(a,b)`

Computes the Kendall's tau-a correlation coefficient between the input vectors (a and b). NaN entries are removed from both.

raeSpearmanCorr

`r=raeSpearmanCorr(x,y,toleratedStandardError)`

This function computes the "random-among-equals" Spearman correlation (RAE-Spearman r), which is the Pearson correlation applied to ranks with random ranks assigned to sets of entries in either variable that have equal values. In case there are no ties, the RAE-Spearman correlation is equal to the conventional Spearman correlation. If there are ties, then the RAE-Spearman correlation uses a random ranks within each equality class (rather than average ranks as in the Spearman correlation).

This modification is motivated by the scenario of comparing model predictions to data, where some models predict ties. The Spearman (and also the Pearson and Kendall tau b and tau c) grants an advantage to models predicting ties over models predicting at chance or slightly better than chance for the same pairs of entries. As a result, these correlation coefficients grant a higher score to models that avoid detailed predictions by predicting ties over models that make more accurate and precise predictions. A simplified model can beat the true model when both are compared to noisy data. The Kendall tau-a does not have this drawback, but it has time complexity $O(n^2)$, where n is the number of entries, making it ill-suited for extensive permutation or bootstrap testing of complex model predictions.

The RAE-Spearman, like the Kendall tau-a, penalizes the prediction of ties by replacing the ties with random guesses. Like the tau-a it rewards a model (in terms of the expected value of the r) for predicting the direction of a pair of entries whenever it has *any information* about the pair of entries (i.e. whenever the probability of a correct guess is larger than 0.5). Both the RAE-Spearman correlation and Kendall's tau-a penalize ties as much as guessing.

The RAE-Spearman has the advantage of being faster to compute than the Kendall tau-a. Its time complexity is O(n*log(n)) (because sorting is O(n*log(n)) and the Pearson correlation is O(n)). It has the disadvantage of being nondeterministic, because random ranks are chosen. The parameter `nReps` offers a simple remedy, controlling how many times the RAE-Spearman is computed and results averaged.

categoricalRDM

`[binRDM, nCatCrossingsRDM]=categoricalRDM(categoryVectors,figI,monitor)`

If given a single vector of category indices, this function returns a binary representational dissimilarity matrix (`binRDM`) containing a zero for each pair of conditions falling into the same category, and a one for each pair of conditions falling into different categories.

Alternatively, if given a set of column vectors that define categories, this function returns a binary RDM indicating the condition pairs straddling at least one category boundary. A 0 indicates that the dissimilarity at that location is between two conditions that are within the same category on all category vectors. A 1 indicates that the dissimilarity is between two conditions that span separate categories according to at least one of the category vectors.

The additional return value `nCatCrossingsRDM` contains a count of the number of category boundaries that divide each pair of conditions. This number ranges from 0 (both conditions in the same category according to all category vectors) to m, the width of category vectors (the conditions are in different categories according to all category vectors).

squareRDM

`RDM=squareRDM(RDM)`

converts RDM to square form, if they are in square form already they are vectorized using the lower triangle (matlab squareform convention) and re-squared, thus fixing inconsistencies between upper and lower triangle (the lower is used).

squareRDMs

`RDMs=squareRDMs(RDMs_ltv)`

converts set of row-vector `RDMs_ltv` to square form (despite being rows, RDMs are stacked along the 3rd dimension, just as square RDMs would be. This avoids ambiguity when the `RDMs_ltv` is square and could be either a single RDM or a number of vectorized RDMs.)

`RDMs` may be bare or wrapped with meta-data in a struct object. They will be returned in the same format as passed.

stripNsquareRDMs

`RDMs_squareNbare=stripNsquareRDMs(RDMs)`

Strips and squares a set of RDMs embedded in a structure (specifying names, etc.) to return the bare RDMs stacked along the 3rd dimension (which are assumed to be in a field called `RDM`) or, if RDMs are already bare, they are returned as passed.

wrapAndNameRDMs

`RDMs=wrapAndNameRDMs(RDMs,names)`

Wraps the RDMs in argument RDMs into a structured array and assigns the names in the cell array `names`.

figureMDSArrangement

figureMDSArrangement(RDM, userOptions, localOptions)

This function draws a multidimensional scaling (MDS) arrangement reflecting the dissimilarity structure of the items whose representational dissimilarity matrix is passed in argument RDM. The function can draw the MDS result as an arrangement of text labels (default), colour dots, or icons (e.g. the experimental stimuli or, more generally, icons denoting the experimental conditions). Which ones of these visualizations are produced is controlled by setting the fields of the options arguments.
RDM: the items' dissimilarity matrix as a structure RDM, or in square-matrix or upper-triangular-vector format.
userOptions: The options structure.
userOptions.analysisName: a string which is prepended to the saved files.
userOptions.rootPath: a string describing the root path where files will be saved. (If the directory specified in the path does not exist, it will be created, as will an enclosed "Figures" directory.
userOptions.criterion: The criterion which will be minimised to optimise the MDS arrangement (defaults to metric stress).
localOptions: optional structure whose fields control which visualizations are produced and provide the required additional information. Each of the fields are also optional. If localOptions is not passed or none of the fields are set, the MDS arrangement will be drawn using text labels, where each label is a number indexing an activity pattern in the order defined by the RDM.

The key fields are:

localOptions.figI_textLabels: Figure index for visualization as text-label arrangement (defaults to 1).

localOptions.figI_catCols: Figure index for visualization as colour-dot arrangement, where the colours code for category.

This visualization is omitted if this argument is missing.

localOptions.figI_icons: Figure index for visualization as an icon arrangement. This visualization is omitted if this argument is missing.

localOptions.figI_shepardPlots: figure index for Shepard plot (a scatterplot relating the dissimilarities in the original space to the distances in the 2D MDS arrangement.

Please note that if the "figI_..." arguments are quadruples instead of single figure indices, then the last three numbers specify the subplot. See the documentation for selectPlot.m for details.

localOptions.MDScriterion: The cost function minimized by the MDS. (Default value is 'metricstress'.) See documentation of mdscale.m for details.

localOptions.rubberbandGraphPlot: Boolean argument. If this is set to 'true', then a rubberband graph is plotted in the background to visualize the distortions incurred by the dimensionality reduction (for details, see Kriegeskorte et al., Neuron, 2008).

Further fields needed for visualization as category-color-coded arrangement of either dots or text labels:
localOptions.contrasts: A matrix, whose columns define categories of items as index vectors (column height == number of items, 1 indicates present, 0 indicates absent). (These category definitions are a special case of a general-linear- model contrast -- hence the name of this argument.)

localOptions.categoryIs: List of integers referring to columns of argument 'contrasts' and thereby selecting which categories of items are to be included in the visualization.

localOptions.categoryColors: nCategories-by-3 matrix, whose rows define the colors as RGB triples. There is one row per category.

localOptions.`categoryLabels`: Text labels for the categories. If these are provided a legend will show what the color-coded categories are.

Further field needed for visualization as icon arrangement:
localOptions.`icons`: The icon images for visualization as an icon arrangement.

`shepardPlot`

`shepardPlot(dissimilarities,disparities,distances,figI,titleString)`

Displays a Shepard plot for the result of multidimensional scaling (MDS).

`dissimilarities`: The dissimilarities to be depicted.

`disparities (optional)`: The corresponding monotonically transformed distances MDS strives to produce in the low-dimensional arrangement.

`distances`: The actual distances of the low-dimensional MDS arrangement.

`selectPlot`

`[hf,ha,figI]=selectPlot(figI)`

If given an integer `figI`, `selectPlot` activates or creates a figure with handle `figI` and sets the background colour to white.
If given a 4x1 or 4x1 vector `figI`, `selectPlot` activates or creates figure `figI(1)`, sets the background colour to white, and further activates the `figI(2:4)` subplot.

`rubberbandGraphPlot`

`rubberbandGraphPlot(coords_xy,distmat,connectionAreaProportionORcolorCoding)`

Given a set of coordinates `coords_xy` (a 2xn matrix with first column for x-ordinates and second column for y-ordinates), the corresponding distance matrix (`distmat`) and a ratio for scaling the areas (`connectionAreaProportionORcolorCoding`, greater values corresponding to larger areas), this function creates a rubberband graph in which points defined by `cords_xy` are connected by "rubberbands" for which the area is proportional to the exact dissimilarity. Like rubber, these lines become thin when "stretched" by MDS distorting distance values. This feature enables displaying the exact dissimilarities in an MDS plot that may be distorted due to dimensionality reduction (see also `shepardPlot.m`).

`plotDotsWithTextLabels`

`plotDotsWithTextLabels(coords_xy, localOptions)`

Plots the set text labels in cell array `textLabels` at the positions `coords_xy` in the current plot in font size `fontSize` in black (by default) or using category colors specified by optional further arguments `categoryColumns`, `categoryColors`, and `categoryLabels`.

`localOptions` is the usual common options struct, with the following fields being used by `plotDotsWithTextLabels`:
`textLabels`: Cell of strings.
`dotColours`: nItems x 3 matrix (one colour per dot).
`fontSize`: Integer specifying the font size of the text.

`categoryColumns`: a vector specifying the category indices. The same integer denotes that the given coordinates belong to the same category and are displayed with the color given in categoryColours field.
`categoryColours`: a matrix specifying the category colours. Category colours are denoted by triples in the same order as defined in the other vectors.
`categoryLabels`: the labels for different categories.

pageFigure

```
h=pageFigure(figI,paperSizeORheightToWidth,proportionOfScreenArea,horPos0123,landscapeFig)
```

This function creates a page figure with figure with specific properties.

plotTextLabels

```
plotTextLabels(coords_xy,textLabels,fontSize,categoryColumns,categoryColors,categoryLabels)
```

Plots the set text labels in cell array `textLabels` at the positions `coords_xy` in the current plot in font size `fontSize` in black (by default) or using category colors specified by optional further arguments `categoryColumns`, `categoryColors`, and `categoryLabels`.

gotoDir

```
gotoDir(varargin)
```

`gotoDir(path, dir)`: Changes the current working directory to path; then to path/`dir`, making it if necessary.

`gotoDir(path)`: Changes the current working directory to `path`, making all required directories on the way.

interleaveRDMs.m

```
RDMs = interleaveRDMs(RDMs, varargin)
```

interleaveRDMs is a function which accepts an arbitrarily-high-dimensional structure of RDMs and recursively "interleaves" the final (non-singleton) dimensions out by shuffling and stacking, finally returning a 1 (or 2, with 1 singleton dimension) dimensional structure of RDMs all ready for display.

vectorizeRDMs

`RDMs_utv=vectorizeRDMs(RDMs)`

Converts a set of RDMs (stacked along the 3rd dimension) to lower-triangular form (set of row vectors).

figureDendrogram

`figureDendrogram(RDM, userOptions, localOptions)`

Generates a dendrogram representation of an RDM and handles the figure according to user preferences.

RDM: The distance matrix basis for the dendrogram. RDM should be in squareform: a [nConditions nConditions]-sized matrix.

UserOptions : The options structure with the following fields:
userOptions.analysisName: A string which is prepended to the saved files.
userOptions.rootPath: A string describing the root path where files will be saved (inside created directories).
userOptions.conditionColours: A [nConditions 3]-sized matrix indicating an [R G B] triple colour for each condition.
userOptions.saveFigurePDF: A Boolean value. If true, the figure is saved as a PDF (defaults to false).
userOptions.saveFigurePS: A Boolean value. If true, the figure is saved as a PS (defaults to false).
userOptions.saveFigureFig: A Boolean value. If true, the figure is saved as a MATLAB .fig file (defaults to false).
userOptions.displayFigures: A Boolean value. If true, the figure remains open after it is created (defaults to true).

localOptions: Further options.

localOptions.linkageType: The Matlab linkage type used for clustering. See Matlab's documentation for "linkage" for more information. (Defaults to 'single'.)

localOptions.colorThreshold: (Defaults to uncoloured.) see Matlab's built in dendrogram.m for the usage of this.

Figures may be exported according to the preferences set in userOptions.

concatenateRDMs

`RDMsOut = concatenateRDMs(RDMs1[, RDMs2[, ...]])`

This function concatenates a number of wrapped RDMs (RDM structures). The main difference between this function and the similar one called `'concatRDMs'` is that this function requires the input to be wrapped RDM structures.

RDMCorrMat

```
corrMat=RDMCorrMat(RDMs,figPlotSpec,type)
```

Returns and optionally displays the correlation matrix (spearman) of a set of `RDMs` (can be square or upper triangle form and wrapped or unwrapped). The matrix is displayed in the figure with a number specified by `figPlotSpec`. `type` indicates the RDM correlation type.

ceilingAvgRDMcorr

```
[ceiling_upperBound, ceiling_lowerBound,
bestFitRDM]=ceilingAvgRDMcorr(refRDMestimates,RDMcorrelationType,monitor)
```

Given a set of reference RDM estimates (e.g. from multiple subjects) in argument `refRDMestimates`, this function estimates upper and lower bounds on the noise ceiling, i.e. the highest average RDM correlation (across the RDM estimates) that the true model's RDM prediction achieves given the variability of the estimates.

The upper bound on the ceiling is estimated by finding the hypothetical model RDM that maximises the average correlation to the reference RDM estimates (using the correlation coefficient specified by argument `RDMcorrelationType`).

For Pearson and Spearman correlation, we have closed-form solutions. For Pearson correlation, the exact upper bound is the average correlation across subjects to the mean RDM computed after z-transforming the dissimilarities in each subject's RDM. For Spearman correlation, the exact upper bound is the average correlation across subjects to the mean RDM computed after rank-transforming the dissimilarities in each subject's RDM.

For Kendall tau-a, there is a closed-form solution for an upper bound on the ceiling utilising the fact that the tau-a correlation distance is proportional to the squared Euclidean distance in pair-relation space. Unfortunately, this upper bound is not tight enough to be useful. We therefore initially estimate the upper bound as the average Kendall tau-a between the mean RDM computed after rank-transforming each RDM, and each subject's RDM. This provides a slight underestimate of the upper bound. We therefore attempt to iteratively optimise the estimate by searching an RDM that yields a higher average tau-a correlation with the single-subject RDMs. In practice, we have observed only minimal improvements. When the RDM space is very high-dimensional, search is difficult and Kendall tau-a takes long to compute, we therefore terminate the search after `historyLength`-many iterations and return the best estimate of the upper bound (which slightly underestimates the true upper bound).

We estimate the lower bound of the ceiling using a leave-one-subject-out cross validation procedure.

Signrank_onesided

```
p = signrank_onesided(x);
```

This function returns the p-value (p) from a one-sided Wilcoxon signed rank test comparing against the input vector, x against zero. The exact method is chosen to get the p-values with greater resolution.

addComparisonBars

`y = addComparisonBars(pairwisePs,cYMax,threshold)`

This function adds the pairwise comparison bars to a figure. Note that Matlab's `'hold on'` should be set before executing this function for it to function as intended. `pairWisePs`: An nxn pairwise comparison matrix. This is supposed to be sorted in descending order of average values (large to small heights) a horizontal bar would be displayed whenever the (one-sided) comparison reaches a threshold. This function adds horizontal lines to the current figure. The only output, y, is the height of the highest bar that is plotted.

`cYMax`: is the height of the lowest bar. This allows the user to specify where the lowest bar would be plotted so that the bars wouldn't interfere with the main plot.
`threshold`: in case `pairwisePs(i,j)` < threshold a line would be drawn between i and j.
Finally the output `y` is the height of the highest horizontal line that is superimposed on the figure.

compactPvalueString

`string=compactPvalueString(p)`

Given a numeric p-value (`p`), this function returns a string (`string`) that expresses it in scientific format.

mat2RGBimage

`RGBim=mat2RGBimage(mat,cols,clims)`

Given a matrix mat, a colormap `cols`, and colorscale-limiting values `clims`, this function returns an RGB image `RGBim` in which the mat is displayed using the colormap, such that the first color in `cols` maps onto matrix value `clims(1)` and the last color in `cols` maps onto `clims(2)`, with a linear scaling in between these values. Matrix values smaller than `clim(1)` are represented by `cols(1,:)` and matrix values larger than `clims(2)` by `cols(end,:)`.

xticklabel_rotate

`hText = xticklabel_rotate(XTick,rot,varargin)`

Input:

`[XTick]`: Vector array of XTick positions & values (numeric) uses current XTick values or XTickLabel cell array by default (if empty).
`[rot]` : Angle of rotation in degrees, 90° by default.
`[XTickLabel]`: Cell array of label strings, empty by default.

`[varargin]`: "Property-value" pairs passed to text generator; e.g. 'interpreter','none, 'Color', 'm', 'Fontweight', 'bold'.

Output:
`hText`: Handle vector to text labels.

Example 1: Rotate existing XTickLabels at their current position by 90°

xticklabel_rotate

Example 2: Rotate existing XTickLabels at their current position by 45° and change font size

```
xticklabel_rotate([],45,[],'Fontsize',14)
```

Example 3: Set the positions of the XTicks and rotate them 90°

```
figure;  plot([1960:2004],randn(45,1)); xlim([1960 2004]
```

xticklabel_rotate([1960:2:2004]);

Example 4: Use text labels at XTick positions rotated 45° without tex interpreter

```
xticklabel_rotate(XTick,45,NameFields,'interpreter','none');
```

Example 5: Use text labels rotated 90° at current positions

```
xticklabel_rotate([],90,NameFields);
```

Note: you cannot re-run `xticklabel_rotate` on the same graph. This function changes the Xtick labels only once.

bootstrapRDMs

```
[realRs bootstrapEs pairwisePs bootstrapRs] =
bootstrapRDMs(bootstrappableReferenceRDMs, candRDMs, userOptions)
```

This function gets a set of reference RDMs and a set of candidate RDMs. Depending on the user's preferences it performs bootstrapping of subject and/or experimental conditions and returns as its output RDM correlations for comparing candidate RDMs nad reference RDMs and also the bootstrap standard deviation for each comparison.

bootstrappableReferenceRDMs: The RDMs to bootstrap. Should be a [nConditions nConditions nSubjects]-sized matrix of stacked squareform RDMs.

candRDMs: The RDMs to test against. Should be an [nConditions nConditions nCandRDMs]-sized matrix where each leaf is one RDM to be tested.

userOptions: The standard options structure, with the following fields:

userOptions.corrType: A string descriptive of the distance measure to be used. (Defaults to 'Spearman'.)

userOptions.nBootstrap: How many bootstrap resamplings should be performed? (Defaults to 1000.)

userOptions.resampleSubjects: Boolean. If true, subjects will be bootstrap resampled. (Defaults to false.)

userOptions.resampleConditions: Boolean. If true, conditions will be resampled. (Defaults to true.)

realRs: The true RDM correlations between the average of the bootstrappableReferenceRDMs and the testRDM.

bootstrapStdE The bootstrap standard error.

relRankIn_includeValue_lowerBound

```
p=relRankIn_includeValue_lowerBound(set,value)
```

This function returns the relative rank of value within set. The relative rank is the proportion of set smaller than value.

simulateDataFiles

```
[betaCorrespondence_true betaCorrespondence_noisy fMRI] = simulateDataFiles(userOptions, simulationOptions)
```

Creates and saves Matlab files for each subject specified in userOptions containing patterns clustered according to simulationOptions, both 'noiseless' and 'noisy'.
betaCorrespondence_true is a set of strings, one per condition. Each refers to the path where the noiseless response pattern files for that condition are stored.
betaCorrespondence_noisy is a set of strings, one per condition. Each refers to the path where the noise-contaminated response patterns for that condition are stored.
fMRI is a structure containing the raw data and the design matrices for both noisy and noiseless patterns.

replaceWildCards

```
varargout = replaceWildcards(stringIn, varargin)
stringOut = replaceWildcards(stringIn[, wildcard1, replacement1, wildcard2, replacement2, ...])
```

This is a function which takes a string "`stringIn`" and (recursively) replaces all occurrences of {`wildcard1`, `wildcard2`, ...} with {`replacement1`, `replacement2`, ...} until no instances of any wildcards remain.

simulateClusteredfMRIData

`[varargout] = simulateClusteredfMRIData(simulationOptions)`

Simulate both a 'true' beta matrix with clustered data (according to `simulationOptions`) and a 'noisy' one contaminated with simulated scanner noise. For a full description on how to define the `simulationOptions`, refer to the function `generateBetaPatterns.m`

generateBetaPatterns

`B = generateBetaPatterns(clusterSpec, nVoxels, centroid)`

Generates a [`nConditions nVoxels`]-sized beta matrix of data where the responses are clustered according to `clusterSpec` (see below). Does not simulate scanner noise --- these simulated responses are "true".

`B`: The beta matrix. A [nConditions nVoxels]-sized matrix of simulated response.

`clusterSpec`: A cell array specification for the clustering of conditions. Clustering of the conditions is specified in the following manner:

- Each cell represents a hierarchy.

- The first entry in a cell is the 'spread' of that level of the hierarchy.

- If the second entry is another cell, it and all following entries (which must also be cells) are sub-hierarchies.

- If the second entry is a number (in which case there must only be two entries), this number represents a number of 'leaves', such that the cell is hierarchically an 'atom'. so we have a well-defined recursive datatype for hierarchies.

For example,

`clusterSpec = {20, {6, {3, 5},{2, 3}}, {4, 7}}`

represents the following hierarchy:

```
             |
  ------------------------ 20
  |                      |
```

```
------------- 6                |
 |            |             ------- 4
----- 3      --- 2          |||||||
|||||        |||            |||||||
 [5]         [3]              [7]
```

`nVoxels`: The number of voxels in the simulated RoI, an integer.
`centroid`: The centre of the clusters.

This is mainly used for the recursion in this function, but a vector of length nVoxels put here will centre the clusters around this point in voxel space. Defaults to the origin.

generateCognitiveModel_fastButTrialsNeedToStartOnVols

```
[cognitiveX,BVstimProt,standardIndexSequence,hirf_ms]=generateCognitiveModel_fastButTrialsNeedToStartOnVols(sequence,stimDur_ms,trialDur_ms,nTRvols_final,TRvol_ms,nSkippedVols,monitor,scaleTrialResponseTo1)
```

This function generates a design matrix of haemodynamic response predictors from a condition sequence. It requires the trial duration to be a multiple of the `TRvol` and the trials to start in register with the volume acquisitions. Highest trial index is assumed to be null (rest or fixation) trial.

Design matrix is generated in milliseconds and then subsampled at the `TRvol` resolution.

temporallySmoothTimeSpaceMatrix

```
Y=temporallySmoothTimeSpaceMatrix(Y,FWHM)
```

This function is used to smooth the time-by-space matrix `Y` with a Gaussian kernel of full width at half maximum `FWHM`.

spatiallySmooth4DfMRI_mm

```
[Y,smoothedYfilename]=spatiallySmooth4DfMRI_mm(Y_OR_Yfilename,volSize_OR_mask,gaussianKernelFWHM_mm,voxSize_mm)
```

This function is used to spatially smooth a 4D fMRI data set stored in variable `Y` in Matlab or in data file `Yfilename.mat` as a time-by-voxel matrix of dimensions specified in triple `volSize` = [xwidth, ywidth, zwidth].

randomPermutation

`indicesOut = randomPermutation(n)`

This function generates a vector of `n` integer numbers from 1 to `n`. The numbers are each used exactly once in the output and are randomly placed in the vector.

gaussian_nk

`g=gaussian_nk(FWHM, nSamples, x, mu, mass)`

Returns a row-vector Gaussian of full width at half maximum `FWHM`. `nSamples` is chosen automatically unless it is passed as an argument. If the other params are unspecified the domain is -(nSampels-1)/2:(nSampels-1)/2 and `nSamples` must be odd, so there is a central sample for the maximum of the Gaussian. The center `mu` is then placed at zero and the `mass` (sum(g)) is set to 1. The unit of the `FWHM` is assumed to be the sampling period (i.e. the interval between adjacent samples), unless the domain `x` is passed. If the domain `x` is passed, it needs to have `nSamples` many points and it will define the units of `FWHM`.

randomlyPermute

`v = randomlyPermute(v)`

Randomly permutes the elements of the input vector.

vec2map

`map=vec2map(vec,mask)`

This function can be used to convert space column vector vec into a spatial map of dimensions of array mask. `mask` is a logical array whose true values must correspond to `vec` in number and order. The returned maps are zero, wherever the mask is false. In a situation where you have a mask defining an RoI (1s inside the RoI, 0s outside), one could use this function to fill the RoI with statistical values from a vector "vec". The result will be a map "map" with 0s outside the original RoI and values taken from vec inside.

map2vec

`vec=map2vec(map,mask)`

This function is used to convert a spatial map of dimensions of array `mask` into space column vector `vec`. `mask` is a logical array whose true values will correspond to `vec` in number and order.

overwritePrompt

```
overwriteFlag = overwritePrompt(userOptions, promptOptions)
```

This is a function which prompts the user, either at the command line or (when it's implemented) via a GUI dialogue box. The user will be notified if they are liable to overwrite pre-existing data and will be given the options to go ahead, to quit, or to skip this section. This also allows an interrupted analysis to be resumed.

`userOptions` : The options structure.

`userOptions.analysisName`: A string which is prepended to the saved files.

`userOptions.rootPath`: A string describing the root path where files will be saved (inside created directories).

`userOptions.forcePromptReply`: A way to automatically reply in a certain way to the overwrite prompt.

`promptOptions` : Further options. For example the user can specify the default option here. Or they can specify the set of file names so that if they do not exist the `overwriteFlag` would be set.

boyntonModel

```
hirf=boyntonModel(res)
```

This function returns a vector containing the haemodynamic impulse response function as estimated by Boynton, Engel, Glover and Heeger (1996) for two subjects' V1. The parameter `res` controls the temporal resolution (time bin width is 1 ms).

constructModelRDMs

```
constructModelRDMs(rawModels, userOptions)
```

`constructModelRDMs` is a function which parses the `rawModels` and saves a structure which is readable by the other toolbox functions.
`rawModels`: Naturally specified models: a structure in which rawModels.(modelName) is the model RDM.

`userOptions`: The standard options structure containing the following fields.

`userOptions.analysisName`: A string which is prepended to the saved files.

`userOptions.rootPath`: A string describing the root path where files will be saved (inside created directories).

`userOptions.ModelColor`: A triple indicating the [R G B] value of the colour which should be used to indicated model RDMs on various diagrams. Defaults to black ([0 0 0]).

The following files are saved by this function:

`userOptions.rootPath/RDMs/`

`userOptions.analysisName_Models.mat`

Contains a structure of model RDMs, one for each of the ones from `rawModels` that is in the form preferred by the toolbox.

showVoxObj

```
showVoxObj(mapORcoords[, figI, subplotTriple, adjustMargin, axisLabels])
```

This function displays a binary voxel object defined by `mapORcoords`

The function has the following input arguments:

`mapORcoords`: this can be a 3D binary map or a list of 3D coordinates (nVoxels by 3).

[`adjustMargin`]: should be left unspecified or set to 1 (default) for normal display. The margin is then adjusted to the bounding box of the object (expanded or reduced), such that there is a 1 voxel margin around the object before upsampling.

[`axisLabels`]: optional structure with fields 'x','y' and 'z', which have string values defining the axis labels.

underscoresToSpaces.m

```
stringORstringInCell=underscoresToSpaces(stringORstringInCell)
```

underscoresToSpaces is a function based on the deunderscore.m. It takes an incoming string or cell array containing strings (`stringORstringInCell`) and replaces all underscores (which are sometimes interpreted by MATLAB as subscript indicators in figures) as spaces (which aren't). It returns the new underscore-replaced string or cell array (`stringORstringInCell`).

addHeading

```
addHeading(heading,figI,x,y)
```

Adds a heading to a figure. The user also has the option of specifying the location of the inserted heading text.

`heading` is a string that is to be added as the figure heading. `figI` is the index of the figure for which the heading will be inserted. `x` and `y` are the position of the text in the figure.

fisherDiscrTRDM

```
[RDM_fdtFolded_ltv, cv2RDM_fdt_sq] = fisherDiscrTRDM(Xa, Ya, Xb, Yb, condPredIs, RDMmask)
```

Computes a lower-triangular vector of Linear Discriminat t-statistics (`RDM_fdtFolded_ltv`) and a folded symmetric LDt RDM (`cv2RDM_fdt_sq`). Inputs to this function are the design matrices and activation matrices for two independent datasets (`a` and `b`). `CondPredIs` is a vector of the indices for which the discriminabilities will be computed (usually 1: number of conditions). `RDMmask` constraints the analysis to the ones that are marked 1 only.

LDt values are based on error covariance matrix. The shrinkage method introduced by Ledolt and Wolf (2013) is used to estimate the covariance matrix.

simulateClusteredfMRIData_fullBrain

```
[B_true, msk, Y_true, fMRI] = simulateClusteredfMRIData_fullBrain(simulationOptions)
```

This function simulates fMRI data with a categorical effect inserted at a specified location. The remaining regions contain noise that is temporally and spatially smooth yet lacks any particular categorical structure. The size of the simulated volume is indicated by the `volumeSize_vox` field and the effect center by `effectCen`. The volume of the simulated effect (within the brain volume) is controlled by `volumeSize_vox`.

`B_true` contains the noiseless betas that simulate the ground truth in the simulated region and zero outside.

`Msk` is a binary ROI. It has the size of the simulated brain, contains ones where the effect is inserted and zeros elsewhere.

`Y_true` is the time series for the voxels within the region where an effect is simulated.

`fMRI` is a structure with the following fields:

`fMRI.B`: The noise contaminated betas

`fMRI.Y`: The noisy time-series

`fMRI.X`: The simulated design matrix

`fMRI.groundTruth`: The response matrix with the structure indicated in simulationOptions.clusterSpec. It contains the response for the simulated region only.

sphericalRelativeRoi

```
relRoi = sphericalRelativeRoi(rad_mm, voxelSize_mm)
```

A function that creates a solid sphere of voxels of radius `rad_mm`.

`rad_mm`: The sphere's radius in millimeters.

`voxelSize_mm`: A triple specifying the voxel size along the three dimensions in the order x, y, z in millimeters.

sphereRelRoi: The voxel sphere as a ROI matrix (nVox by 3), i.e. a list of voxel locations.

Each row [x, y, z] contains the RELATIVE coordinates (with the sphere centered at (0, 0, 0)). This function is particularly useful for displaying spherical searchlight volumes.

addRoiToVol

`vol=addRoiToVol(vol, roi, color, solid)`

This function is used to mark the specified region of interest (roi) to the true-color volume (vol).

Vol: The volume as a stack of true-color slices: X by Y by 3 by Z. The third dimension encodes the color component (red, green, blue). Anatomically, the X axis points to the left, the Y axis to the back of the brain, and the Z axis up.

Roi: A region of interest defined as a set of voxels. roi is a matrix of size # voxels by 3, every row of which specifies a voxel by its ONE-BASED coordinate triple (x,y,z).

Color: Optional red-green-blue triple [r g b] specifying the color. Values range from 0 to 1. Defaults to cyan.

Solid: Optional flag that determines whether the RoI is marked solidly (1, default) or only by its 3D contour (0) or by its 2D (within-slice) contour (2).

If we already have a volume containing colour information and we want to highlight an area (roi) in the volume (vol) with a specific colour; then we indicate the new area by the indices in roi and the new colour in color. A new vol will be returned which has the area highlighted. The colour content of the other voxels (the ones outside roi) remains unchanged in the new vol.

addBinaryMapToVol.m

`vol=addBinaryMapToVol(vol, map, col)`

This function is used to superimpose the binary map (map) to the true-color volume (vol). Where map=1 the corresponding voxel of volume vol is marked in the color col. All other voxels retain their color.

vol: The volume as a stack of true-color slices: X by Y by 3 by Z. The third dimension encodes the color component (red, green, blue). Anatomically, the X axis points to the left, the Y axis to the back of the brain, and the Z axis up.

Map: A statistical map as a 3D array of double-precision floats.

Col: Triple row vector [R G B] specifying the color, in which voxels that contain a 1 in the map (map) are marked in the volume (vol). Values should be element of [0,1].

The function returns the true-color volume (X by Y by 3 by Z) with the binary map superimposed in color col.

map2vol

```
vol=map2vol(map)
```

This function converts a `map` (e.g. statistical t-map) to a true-color volume. It can be helpful for displaying a map using the other function called `showVol.m`.

Note that if the input `map` is a 3D matrix the output would be [x y 3 z] dimensions. The third dimension denotes the colour. Using this function assigns the same value (overall value or the amplitude) for the colour. This makes the displayed volume a greyscale image. If the input matrix has more than 3 dimensions, the function first converts it to a 3D matrix and then proceeds in the explained way.

showVol

```
showVol(volORmap, title, figI, right, skipNslices, nHorPanels, nVerPanels,
labels, labelPos)
```

This function displays a volume as a set of slices.

`Vol`: The volume as a stack of true-color slices: X by Y by 3 by Z. the third dimension encodes the color component (red, green, blue). Anatomically, the X axis points to the left, the Y axis to the back of the brain, and the Z axis up.

`[title]`: String to be used as a title (optional).

`[figI]`: Figure number (optional), generates auto-numbered new figure if this argument is missing or empty.

`[right]`: A string specifying the orientation of the slices. If right is "right", then right is right, i.e. the view onto the slices is from above. If right is "left" (or simply “wrong"), then right is left, i.e. the view onto the slices is from below.

`[skipNslices]`: Optional number of slices to be skipped between slices to be shown. Defaults to 0 (all slices shown).

`Labels`: Structured array of string labels (optional).

`labelPos`: nLabels by 3 matrix of label positions (optional).

paneling

```
[nVerPan nHorPan]=paneling(nPanels,nHorPanOVERnVerPan);
```

specifies the number of Horizontal panels (`nHorPan`) and the number of vertical panels (`nVerPan`) given the total number of panels and the aspect ratio of the figure (number of horizontal panels/number of vertical panels). This may be used to find the suitable number of subplots when displaying multiple plots simultaneously.

scale01

```
Xscaled=scale01(X,range)
```

This function linearly scales its input (matrix `X`) into the range [0,1]. If `range` is given, `min(range)` and `max(range)` define the scaling and shifting, rather than `min(X(:))` and `max(X(:))`.

covdiag

```
sigma=covdiag(x)
```

Shrinks the covariance matrix from the input data , `x`, towards diagonal matrix

`x` (t*n): t iid observations on n random variables

`sigma` (n*n): invertible covariance matrix estimator

fMRIDataMasking

```
[varargout] = fMRIDataMasking(fullBrainVols, binaryMasks_nS,
betaCorrespondence, userOptions);
```

`fMRIDataMasking` is a function which takes a full set of fMRI brain scans and masks them according to the binary masks specified by the user (if any). If no masks are specified then no masking is done.

[`responsePatterns` =] `fMRIDataMasking`(`fullBrainVols`, `binaryMasks_nS`, `userOptions`)

`fullBrainVols` --- The unmasked beta (or t) images.

`fullBrainVols`.(`subject`) is a [`nVoxel` `nCondition` `nSession`]-sized matrix. The order of the voxels is that given by reshape or Matlab's colon operator and is consistent across subjects and binary mask definitions.

`binaryMasks_nS` --- The native- (subject-) space masks.

`binaryMasks_nS.(subject).(mask)` is a [x y z]-sized binary matrix (the same size as the native-space 3D beta images.

`betaCorrespondence` --- The array of beta filenames. `betas(condition, session).identifier` is a string which refers to the filename (not including path) of the SPM beta image. (Or, if not using SPM, just something, as it's used to determine the number of conditions and sessions.)

`userOptions` --- The options struct.

`userOptions.analysisName`: a string which is prepended to the saved files.

userOptions.rootPath: a string describing the root path where files will be saved (inside created directories).

userOptions.subjectNames: a cell array containing strings identifying the subject names. Defaults to the fieldnames in fullBrainVols.

userOptions.maskNames: a cell array containing strings identifying the mask names. Defaults to the fieldnames of the first subject of binaryMasks_nS.

The following files are saved by this function:

userOptions.rootPath/ImageData/userOptions.analysisName_responsePatterns.mat

It contains the masked brains in a struct such that responsePatterns.(mask).(subject) is a [nMaskedVoxels nConditions nSessions] matrix.

userOptions.rootPath/Details/userOptions.analysisName_fMRIDataMasking_Details.mat This file contains the userOptions for this execution of the

fMRIMaskPreparation

```
[binaryMasks_nS =] fMRIMaskPreparation(userOptions)
```

fMRIMaskPreparation will load SPM-defined masks from a directory specified in userOptions and will save a struct containing binary mask matrices.

userOptions --- The options struct.

userOptions.analysisName: a string which is prepended to the saved files. userOptions.rootPath: a string describing the root path where files will be saved (inside created directories).

userOptions.maskPath: a string describing the path to the location of the files for the definition of RoI masks.

userOptions.subjectNames: a cell array containing strings identifying the subject names.

userOptions.maskNames: a cell array containing strings identifying the mask names.

The following files are saved by this function:

userOptions.rootPath/ImageData/userOptions.analysisName_Masks.mat

Contains the raw beta images in a struct such that binaryMasks_nS.(subject).(mask) is a [x y z]-sized binary matrix.

userOptions.rootPath/Details/userOptions.analysisName_fMRIMaskPreparation_Dgetails.mat

Contains the userOptions for this execution of the function and a timestamp.

fMRISearchlight

```
[rMaps_sS, maskedSmoothedRMaps_sS, searchlightRDMs[, rMaps_nS, nMaps_nS] =]
fMRISearchlight(fullBrainVols,binaryMasks_nS,models,betaCorrespondence,
userOptions);
```

`fMRISearchlight` is a function which takes some full brain volumes of data, some binary masks and some models and perfoms a searchlight in the data within each mask, matching to each of the models. Saved are native-space r-maps for each model.

The input arguments are:

`fullBrainVols` : The unmasked beta (or t) images. `fullBrainVols.(subject)` is a `[nVoxel nCondition nSession]`-sized

matrix. The order of the voxels is that given by reshape or (:).

`binaryMasks_nS` --- The native- (subject-) space masks.

`binaryMasks_nS.(subject).(mask)` is a [x y z]-sized binary matrix (the same size as the native-space 3D beta images.

`models` --- A stack of model RDMs in a structure. `models` is a [1 `nModels`] structure with fields:

`RDM`

`name`

`color`

`betaCorrespondence` : The array of beta filenames. `betas(condition, session).identifier` is a string which refers to the filename (not including path) of the SPM beta image. (Or, if not using SPM, just something, as it's used to determine the number of conditions and sessions.) Alternatively, this can be the string 'SPM', in which case the SPM metadata will be used to infer this information, provided that `userOptions.conditionLabels` is set, and the condition labels are the same as those used in SPM.

`userOptions` --- The options struct.

`userOptions.analysisName`: a string which is prepended to the saved files.

`userOptions.rootPath`: a string describing the root path where files will be saved (inside created directories).

`userOptions.subjectNames`: a cell array containing strings identifying the subject names. Defaults to the fieldnames in fullBrainVols.

`userOptions.maskNames`: a cell array containing strings identifying the mask names. Defaults to the fieldnames of the first subject of binaryMasks_nS.

`userOptions.voxelSize`: A tripple consisting of the [x y z] dimensions of each voxel in mm.

userOptions.structuralsPath: A string which contains the absolute path to the location of the structural images and the normalisation warp definition file. It can contain the following wildcards which would be replaced as indicated:

[[subjectName]]

To be replaced with the name of each subject where appropriate.

The following files are saved by this function:

userOptions.rootPath/Maps/userOptions.analysisName_fMRISearchlight_Maps.mat

Contains the searchlight statistical maps in struct so that ps_nS.(modelName).(subject).(maskName), rMaps_sS.(modelName).(subject).(maskName),

maskedSmoothedRMaps_sS.(modelName).(subject).(maskName)

and nMaps_nS.(modelName).(subject).(maskName) contain the appropriate data.

userOptions.rootPath/RDMs/userOptions.analysisName_fMRISearchlight_RDMs.mat

Contains the RDMs for each searchlight so that searchlightRDMs.(subject)(:, :, x, y, z) is the RDM.

userOptions.rootPath/Details/userOptions.analysisName_fMRISearchlight_Details.mat

Contains the userOptions for this execution of the function and a timestamp.

fisherTransform

```
setOut = fisherTransform(setIn, fakeIt)
```

This function computes a Fisher transform on the input vector (setIn). If the input is 1, the Fisher transform will be infinite. There are cases where the user opts to apply Fisher transform to all the correlation values (with the rationale of making the r distribution more Gaussian hence more appropriate for group-level parametric analysis). In these cases, applying this transform can result in NaN values where there is a correlation of 1 (very rare scenario). Setting the fakeIt argument to 1 replaces the Fisher transform of 1 (default: Inf) with 1/eps where eps is the smallest number defined in Matlab.

`image_thr`

```
image_thr(im,thr)
```

This function takes an image (`im`) and a threshold (`thr`) as its input. It thresholds the image and displays the values *below* the threshold in red. The other values are shown in grayscale. It doesn't return any argument and plots the thresholded matrix in the current figure.

`removeSpaces`

```
stringOut = removeSpaces(stringIn);
```

This function removes all spaces from the input string.

`upSample`

```
largerMap=upSample(map,factor)
```

This function upsamples the input matrix `map` by repeating each value `factor` times. Note that this function does NOT do any interpolation or extrapolation. It upsamples `map` by repeating each matrix element in all dimensions. The function outputs the upsampled matrix.

`mask2roi`

```
roi=mask2roi(mask)
```

Gives the coordinates of the marked points in an input matrix `mask`. This function assumes that any non-zero entries are part of the RoI. For example when the input is a 3D binary mask, the output (`roi`) would be triples containing the coordinates of the points (in matrix space) where the binary mask is set to 1. This function is appropriate for 3D binary masks. For higher-dimensional matrices, it discards the coordinates that are greater than 3. For lower-dimensional maps (e.g. 2D), it returns the mask coordinates in the appropriate space (e.g. x and y coordinates). Note that in cases where the input `mask` is not binary, the function returns the coordinates for all non-zero entries.

`getDataFromSPM`

```
betas = getDataFromSPM(userOptions)
```

`getDataFromSPM` is a function which will extract from the SPM metadata the correspondence between the beta image filenames and the condition and session number.

`betas`: The array of info: a structure with nConditions x nSessions entries. For a particular condition index (condI) and session index (sessI),betas(condI,sessI).identifier is a string that refers to the filename (not including path) of the SPM beta image.

`userOptions`: The options structure containing the following fields:

`userOptions.subjectNames`: A cell array containing strings identifying the subject names.

`userOptions.betaPath`: A string which contains the absolute path to the location of the beta images. It can contain the following wildcards which would be replaced as indicated:

"[[subjectName]]": To be replaced with the contents of subject.

"[[betaIdentifier]]": To be replaced by filenames returned in betas.

`userOptions.conditionLabels`: A cell array containing the names of the conditions in the experiment. Here, these will be used to find condition response beta predictor images (these names should be the same as the ones used for SPM first level analysis).

TOOLBOX CODE DEPENDENCIES

This section lists the top level dependency of the toolbox scripts:

List of engines

- addBinaryMapToVol
- addComparisonBars
- addHeading: pageFigure
- addImageSequenceToAxes
- addRoiToVol
- averageRDMs_subjectSession
- betaCorrespondence
- bootstrapRDMs: setIfUnset, rankCorr_Kendall_taua, vectorizeRDMs, raeSpearmanCorr
- boyntonModel
- brightness
- categoricalRDM: concatRDMs, showRDMs
- ceilingAvgRDMcorr: vectorizeRDMs, unwrapRDMs, showRDMs, rankCorr_Kendall_taua
- colorScale
- compactPvalueString
- concatenateRDMs: interleaveRDMs
- concatRDMs: wrapRDMs
- concatRDMs_unwrapped
- constructModelRDMs: underscoresToSpaces, setIfUnset, gotoDir
- covdiag
- defineUserOptions
- deunderscore
- exportCurrentFigAsPDF: setPapertoFigPos
- exportCurrentFigAsPostScript: setPapertoFigPos
- exportfig
- FDRthreshold
- figureDendrogram: vectorizeRDM, squareRDM, selectPlot, handleCurrentFigure, gotoDir
- figureMDSArrangement: unwrapRDMs, squareRDMs, shepardPlot, selectPlot, rubberbandGraphPlot, plotDotsWithTextLabels, setIfUnset, pageFigure, plotTextLabels, handleCurrentFigure, gotoDir, deunderscore
- fisherDiscrTRDM: squareRDMs, covdiag
- fisherTransform
- fMRIDataMasking: getDataFromSPM, overwritePrompt, gotoDir, setIfUnset
- fMRIMaskPreparation: gotoDir, overwritePrompt, replaceWildcards
- gaussian_nk
- generateBetaPatterns
- generateCognitiveModel_fastButTrialsNeedToStartOnVols: boyntonModel, pageFigure
- getDataFromSPM: replaceWildCards
- gotoDir
- handleCurrentFigure: exportCurrentFigAsPDF, exportCurrentFigAsPostscript, exportfig, setIfUnset
- image_thr: mat2RGBimage, colorScale
- interleaveRDMs
- map2vec
- map2vol
- mask2roi
- mat2RGBimage
- overwritePrompt
- pageFigure

- paneling
- plotDotsWithTextLabels: setIfUnset, deunderscore
- plotTextLabels: deunderscore
- raeSpearmanCorr
- randomlyPermute: randomPermutation
- randomPermutation
- rankCorr_Kendall_taua
- rankTransform
- rankTransform_equalsStayEqual
- RDMcolormap: brightness, colorScale
- RDMCorrMat: vectorizeRDMs, unwrapRDMs, rankCorr_Kendall_taua, selectPlot, colorScale
- relRankIn_includeValue_lowerBound
- removeSpaces
- replaceWildcards
- rubberbandGraphPlot: vectorizeRDM
- scale01
- selectPlot
- setIfUnset
- setPapertoFigPos
- shepardPlot: selectPlot, vectorizeRDM, vectorizeRDMs
- showRDMs: RDMcolormap, unwrapRDMs, rankTransform_equalsStayEqual, paneling, deunderscore, rankTransform, squareRDM, addImageSequenceToAxes, scale01
- showVol: map2vol
- showVoxObj: upSample
- signrank_onesided
- simulateClusteredfMRIData: generateBetaPatterns, randomlyPermute, generateCognitiveModel_fastButTrialsNeedToStartOnVols, spatiallySmooth4DfMRI_mm, temporallySmoothTimeSpaceMatrix
- simulateClusteredfMRIData_fullBrain: generateBetaPatterns, randomlyPermute, spatiallySmooth4DfMRI_mm, temporallySmoothTimeSpaceMatrix
- simulateDataFiles: replaceWildcards, simulateClusteredfMRIData, gotoDir, overwritePrompt
- spatiallySmooth4DfMRI_mm: vec2map, map2vec
- sphericalRelativeRoi
- squareRDM: vectorizeRDM
- squareRDMs: squareRDM, vectorizeRDM, wrapRDMs, unwrapRDMs
- stripNsquareRDMs: squareRDM, squareRDMs
- temporallySmoothTimeSpaceMatrix: gaussian_nk
- underscoresToSpaces
- unwrapRDMs
- upSample
- vec2map
- vectorizeRDM
- vectorizeRDMs: unwrapRDMs, wrapRDMs, vectorizeRDM
- wrapAndNameRDMs: wrapRDMs
- wrapRDMs
- xticklabel_rotate

List of modules

- MDSConditions: setIfUnset, removeSpaces, interleaveRDMs, figureMDSArrangement
- dendrogramConditions: setIfUnset, interleaveRDMs, removeSpaces, figureDendrogram
- pairwiseCorrelateRDMs: setIfUnset, concatenateRDMs, RDMCorrMat, colorScale, handleCurrentFigure, gotoDir

- MDSRDMs: setIfUnset, concatenateRDMs, fRDMCorrMat, figureMDSArrangement
- compareRefRDM2candRDMs: vectorizeRDMs, vectorizeRDM, stripNsquareRDMs, pageFigure, mat2RGBimage, relRankIn_includeValue_lowerBound, rankCorr_Kendall_taua, raeSpearmanCorr, ceilingAvgRDMcorr, signrank_onesided, FDRthreshold, compactPvalueString, addComparisonBars, xticklabel_rotate, randomPermutation, handleCurrentFigure, deunderscore, colorScale, FDRthreshold
- fMRIDataPreparation: replaceWildcards, overwritePrompt, gotoDir,
- spm_read_vols, spm_vol
- constructRDMs: setIfUnset, overwritePrompt, gotoDir
- figureRDMs: setIfUnset, interleaveRDMs, showRDMs, gotodir, handleCurrentFigure
- searchlightMapping_fMRI: vectorizeRDMs, vectorizeRDM, unwrapRDMs, showVoxObj, showVol, setIfUnset, map2vol, fisherTransform, addBinaryMapToVol
- fMRISearchlight: addBinaryMapToVol, fisherTransform, getDataFromSPM, gotoDir, map2vol, overwritePrompt, replaceWildCards, setIfUnset, showVol, showVoxObj, unwrapRDMs, vectorizeRDM, vectorizeRDMs

Total number of m files for Engines and Modules for this release: 97 files. This documentation will be included with the toolbox download, and much of the technical information is reproduced as help text for the Matlab functions themselves.
