# Supplementary material for: A Toolbox for Representational Similarity Analysis
Source: PLoS Comput Biol. 2014 Apr 17;10(4):e1003553. doi: 10.1371/journal.pcbi.1003553 (PMC3990488; doi:10.1371/journal.pcbi.1003553)
Supplement: Text S1 — The supplementary materials (additional text) for the manuscript. (DOCX) [file pcbi.1003553.s007.docx]

Supplementary materials

# A toolbox for representational similarity analysis

Nili H, Wingfield C, Walther A, Su L, Marslen-Wilson W, Kriegeskorte N

Getting started with the toolbox

The toolbox folder (software S1) has a number of built-in, ready-to-use demo files that can serve as a prototype for the user’s analysis. To run the demos and get figures similar to those in the paper, the user should proceed as follows:

(1) Download the toolbox from here:
<http://www.mrc-cbu.cam.ac.uk/methods-and-resources/toolboxes/license/>

(2) Save a local copy of the RSAtoolbox folder.

(3) Open Matlab and set the current directory to the *Demo* subfolder of the toolbox folder
(..\RSAtoolbox\Demos)

1. Run DEMO1_RSA_ROI_simulatedAndRealData.m for a demonstration of ROI-based analysis using the toolbox. It simulates RDMs, analyzes them with RSA, and reproduces the results from Figures 2-5 of the main paper.
2. Run DEMO2_RSA_ROI_sim.m for a demonstration of the ROI-based RSA on simulated fMRI data. This script will familiarize the user with the pipeline for analyzing fMRI data.
3. Run DEMO3_LDt_sim.m for a demonstration of the computation of LD-*t* RDMs and associated inference procedures. Running the script reproduces Figure S5.
4. Run DEMO4_RSAsearchlight_sim.m for a demonstration of the searchlight analysis on simulated fMRI data. Running this script reproduces Figure S3.

The first three demos take a few minutes to run. The searchlight demo can take hours the first time it is run, because it needs to simulate data for the whole brain in multiple subjects. The searchlight analysis of the simulated data takes only a couple of minutes per subject on a modern workstation.

Toolbox modules

The toolbox contains “*recipes*” (i.e. top-level scripts) that implement the previously described analysis steps on fMRI response patterns. For the *recipes* (located in the “*Recipe*” directory) to work, all the user has to do is to define the model RDMs to be included in the analysis and to complete the information in the Matlab script called *projectOptions*. Once the project options have been specified, the *Recipe* function can be executed and results are displayed and saved in specified directories. The *projectOptions* contains information about the data structure (e.g. where the ROI masks or the response patterns are stored) and also analysis settings, including the response-pattern dissimilarity measure, and inference settings.

The table below gives the names and descriptions of the key functions of the toolbox. The right column specifies the analysis step (main paper text) to which the function contributes.

**Table S1: Key functions of the RSA toolbox**

| **function name** | **description** | **step** |
| --- | --- | --- |
| constructRDMs | takes the data matrix (response patterns for all experimental conditions) and computes RDMs from it | 1 |
| figureRDMs | displays a number of RDMs | 1 |
| dendrogramConditions | generates a text-labeled dendrogram for the input RDMs | 1 |
| MDSConditions | generates an MDS or t-SNE arrangement of the stimuli (using stimulus icons or colored dots) for the input RDMs | 1 |
| pairwiseCorrelateRDMs | given a number of RDMs, this module will compute and visualize an RDM correlation matrix (comparing each RDM to each other RDM) | 2 |
| MDSRDMs | draws an MDS arrangement of the RDMs | 2 |
| compareRefRDM2candRDMs | statistical inference function that compares a reference RDM to multiple candidate RDMs using a variety of frequentist nonparametric tests | 3 |

Key function for statistical inference

The key function for statistical inference in the toolbox is compareRefRDM2candRDMs.m. This function implements all inference procedures described in the paper. Here we describe these procedures in greater detail. We first explain the general functionality, with some redundancy to the main paper. We then define the usage and all inputs and outputs of the function in detail. The text below is identical to the help text of compareRefRDM2candRDMs.m, but Figure S1 has been added to give an overview of the statistical inference methods, the circumstances under which each is available, and the default choices.

General purpose

The function compareRefRDM2candRDMs.m compares a reference RDM to multiple candidate RDMs. For example, the reference RDM could be a brain region's RDM and the candidate RDMs could be multiple computational models. Alternatively, the

reference RDM could be a model RDM and the candidate RDMs could be multiple brain regions' RDMs. More generally, the candidate RDMs could include both model RDMs and RDMs from brain regions, and the reference RDM could be either a brain RDM or a model RDM. In all these cases, one reference RDM is compared to multiple candidates.

**<Figure S1 here>**

Testing RDM correlations

The function compares the reference RDM to each of the candidates, tests each candidate for significant RDM correlation (test dependent on input data and userOptions) and presents a bar graph of the RDM correlations in descending order from left to right (best candidates on the left) by default, or in the order in which the candidate RDMs are passed. In addition, pairwise comparisons between the candidate RDMs are performed to assess, for each pair of candidate RDMs, which of the two RDMs is more consistent with the reference RDM. A significant pairwise difference is indicated by a horizontal line above the corresponding two bars. Each bar comes with an error bar, which indicates the standard error, estimated by the same procedure as is used for the pairwise candidate comparisons (dependent on input data and userOptions, see below).

Statistical inference on the correlation between the reference RDM and each candidate RDM is performed using a one-sided Wilcoxon signed-rank across subjects by default. When the number of subjects is insufficient (<12), or when requested in userOptions, the test is instead performed by condition-label randomisation. By default, the false-discovery rate is controlled for these tests across candidate models. When requested in userOptions, the familywise error rate is controlled instead.

Comparisons between candidate RDMs

For the comparisons between candidate RDMs as well, the inference procedure depends on the input data provided and on userOptions. By default, a signed-rank test across repeated measurements of the RDMs (e.g. multiple subjects or sessions) is performed. Alternatively, these tests are performed by bootstrapping of the subjects and/or conditions set. Across the multiple pairwise comparisons, the function controls the familywise error rate (Bonferroni method) or the false-discovery rate [1].

Ceiling upper and lower bounds

If multiple instances of the reference RDM are passed (typically representing estimates for multiple subjects), a ceiling estimate is indicated by a gray transparent horizontal bar. The ceiling is the expected value, given the noise (i.e. the variability across subjects), of the average correlation of the true model's RDM with the single-subject RDMs. The upper and lower edges of the ceiling bar are upper- and lower-bound estimates for the unknown true ceiling. The upper bound is estimated by computing the correlation between each subject's RDM and the group-average RDM. The lower bound is estimated similarly, but using a leave-one-out approach, where each subject's RDM is correlated with the average RDM of the other subjects' RDMs. When Pearson correlation is chosen for comparing RDMs (userOptions.RDMcorrelationType), the RDMs are first z-transformed. When Spearman correlation is chosen, the RDMs are first rank-transformed. When Kendall's tau a is chosen, an iterative procedure is used. See main paper for a full motivation for the ceiling estimate and for an explanation of why these are useful upper- and lower-bound estimates. (See also below, under *(5) Estimating the upper bound on the noise ceiling for the RDM correlation*.)

Usage

stats_p_r = compareRefRDM2candRDMs(refRDM, candRDMs[, userOptions])

Arguments

refRDM

The reference RDM, which can be a square RDM or lower-triangular- vector RDM, or a wrapped RDM (structure specifying a name and colour for colour-coding of the RDM in figures). refRDM may also be a set of independent estimates of the same RDM (square matrices or lower-triangular vectors stacked along the third dimension or a structured array of wrapped RDMs), e.g. an estimate for each of multiple subjects or sessions, which are then used for random-effects inference.

candRDMs

A cell array with one cell for each candidate RDM. The candidate RDMs can be square or lower-triangular-vector RDMs or wrapped RDMs. Each candidate RDM may also contain multiple independent estimates of the same RDM, e.g. an estimate for each of multiple subjects or sessions. These can be used for random-effects inference if all candidate RDMs have the same number of independent estimates, greater than or equal to 12. However, if refRDM contains 12 or more independent estimate of the reference RDMs, then random-effects inference is based on these and multiple instances of any candidate RDMs are replaced by their average. In case the dissimilarity for a given pair of conditions is undefined (NaN) in any candidate RDM or in the reference RDM, that pair is set to NaN in all RDMs and treated as a missing value. This ensures that comparisons between candidate RDMs are based on the same set of dissimilarities.

userOptions.RDMcorrelationType

The correlation coefficient used to compare RDMs. This is 'Spearman' by default, because we prefer not to assume a linear relationship between the distances (e.g. when a brain RDM from fMRI is compared to an RDM predicted by a computational model). Alternative definitions are 'Kendall_taua' (which is appropriate whenever categorical models are tested) and 'Pearson'. The Pearson correlation coefficient may be justified when RDMs from the same origin (e.g. multiple computational models or multiple brain regions measured with the same method) are compared. For more details on the RDM correlation type, see main paper and Figure S2.

userOptions.RDMrelatednessTest

'subjectRFXsignedRank' (default): Test the relatedness of the reference RDM to each candidate RDM by computing the correlation for each subject and performing a one-sided Wilcoxon signed-rank test against the null hypothesis of 0 correlation, so as to test for a positive correlation. (Note that multiple independent measurements of the reference or candidate RDMs could also come from repeated measurements within one subject. We refer to the instances as “subjects”, because subject random-effects inference is the most common case.)

'randomisation': Test the relatedness of the reference RDM to each candidate RDM by randomising the condition labels of the reference RDM, so as to simulate the null distribution for the RDM correlation with each candidate RDM. In case there are multiple instances of the reference or candidate RDMs, these are first averaged.

'conditionRFXbootstrap': Test the relatedness of the reference RDM to each candidate RDM by bootstrapping the set of conditions (typically: stimuli). For each bootstrap sample of the conditions set, a new instance is generated for the reference RDM and for each of the candidate RDMs. Because bootstrap resampling is resampling with replacement, the same condition can appear multiple times in a sample. This entails 0 entries (from the diagonal of the original RDM) in off-diagonal positions of the RDM for a bootstrap sample. These zeros are treated as missing values and excluded from the dissimilarities, across which the RDM correlations are computed. The p value for a one-sided test of the relatedness of each candidate RDM to the reference RDM is computed as the proportion of bootstrap samples with a zero or negative RDM correlation. This test simulates the variability of the estimates across condition samples and thus supports inference generalising to the population of conditions (or stimuli) that the condition sample can be considered a random sample of. Note that basic bootstrap tests are known to be slightly optimistic.

'subjectConditionRFXbootstrap': Bootstrap resampling is simultaneously performed across both subjects and conditions. This simulates the greater amount of variability of the estimates expected if the experiment were repeated with a different sample of subjects and conditions. This more conservative test attempts to support inference generalising across both subjects and stimuli (to their respective populations). Again, the usual caveats for basic bootstrap tests apply.

'none': Omit the test of RDM relatedness.

userOptions.RDMrelatednessThreshold

The significance threshold (default: 0.05) for testing each candidate RDM for relatedness to the reference RDM. Depending on the choice of multiple testing correction (see next userOptions field), this can be the expected false-discovery rate, the familywise error rate, or the uncorrected p threshold.

userOptions.RDMrelatednessMultipleTesting

'FDR' (default): Control the false-discovery rate across the multiple tests (one for each candidate RDM). With this option, userOptions.RDMrelatednessThreshold is interpreted to specify the expected false-discovery rate, i.e. the expected proportion of candidate RDMs falsely declared significant among all candidate RDMs declared significant.

'FWE': Control the familywise error rate. When the condition-label randomisation procedure is selected to test RDM relatedness, then randomisation is used to simulate the distribution of maximum RDM correlations across all candidate RDMs. This method is more powerful than Bonferroni correction when there are dependencies among candidate RDMs. If another test is selected to test RDM relatedness, the Bonferroni method is used. In either case, userOptions.RDMrelatednessThreshold is interpreted as the familywise error rate, i.e. the probability of getting any false positives under the omnibus null hypothesis that all candidate RDMs are unrelated to the reference RDM.

'none': Do not correct for multiple testing (not recommended). With this setting, userOptions.RDMrelatednessThreshold is interpreted as the uncorrected p threshold.

userOptions.candRDMdifferencesTest

'subjectRFXsignedRank' (default, data permitting): For each pair of candidate RDMs, perform a statistical comparison to determine which candidate RDM better explains the reference RDM by using the variability across subjects of the reference or candidate RDMs. The test is a two-sided Wilcoxon signed-rank test of the null hypothesis that the two RDM correlations (refRDM to each of the candidate RDMs) are equal. This is the default test when multiple instances of the reference RDM (typically corresponding to multiple subjects) or a consistent number of multiple instances of each candidate RDMs is provided and the number of multiple instances is 12 or greater. This test supports inference generalising to the population of subjects (or repeated measurements) that the sample can be considered a random sample of.

'subjectRFXbootstrap': For each pair of candidate RDMs, perform a two-sided statistical comparison to determine, which candidate RDM better explains the reference RDM by bootstrapping the set of subjects. For each bootstrap sample of the subjects set, the RDMs are averaged across the bootstrap sample and the difference between the two RDM correlations (refRDM to each of the candidate RDMs) is computed. The p value is estimated as the proportion of bootstrap samples further in the tails (symmetrically defined for a two-sided test) than 0. This test simulates the variability of the estimates across subject samples and thus supports inference generalising to the population of subjects (or repeated measurements) that the sample can be considered a random sample of. The usual caveats for basic bootstrap tests apply.

'conditionRFXbootstrap': For each pair of candidate RDMs, perform a two-sided statistical comparison to determine which candidate RDM better explains the reference RDM by bootstrapping the set of conditions (typically: stimuli). For each bootstrap sample of the conditions set, a new instance is generated for the reference RDM and for each of the candidate RDMs. Because bootstrap resampling is is resampling with replacement, the same condition can appear multiple times in a sample. This entails 0 entries (from the diagonal of the original RDM) in off-diagonal positions of the RDM for a bootstrap sample. These zeros are treated as missing values and excluded from the dissimilarities, across which the RDM correlations are computed. The p value for the two-sided test of the difference for each pair of candidate RDMs is computed as for the setting subjectRFXbootstrap (see above). This test simulates the variability of the estimates across condition samples and thus supports inference generalising to the population of conditions (typically stimuli) that the condition sample can be considered a random sample of. Again, the usual caveats for bootstrap tests apply.

'subjectConditionRFXbootstrap': Bootstrap resampling is simultaneously performed across both subjects and conditions. This simulates the greater amount of variability of the estimates expected if the experiment were repeated with a different sample of subjects and conditions. This more conservative test attempts to support inference generalising across both subjects and stimuli (to their respective populations. However, the usual caveats for bootstrap tests apply.

'none': Omit the pairwise tests of candidate RDMs comparing their relative ability to explain the reference RDM.

userOptions.candRDMdifferencesThreshold

The significance threshold for comparing each pair of candidate RDMs in terms of their relatedness to the reference RDM. Depending on the choice of multiple testing correction (see next userOptions field), this can be the expected false-discovery rate, the familywise error rate, or the uncorrected p threshold.

userOptions.candRDMdifferencesMultipleTesting

'FDR': Control the false-discovery rate across the multiple tests (one for each candidate RDM). With this option, userOptions.candRDMdifferencesThreshold is interpreted to specify the expected false-discovery rate, i.e. the expected proportion of pairs of candidate RDMs falsely declared significantly different among all pairs of candidate RDMs declared significantly different (in their relatedness to the reference RDM).

'FWE': Control the familywise error rate. With this option, the Bonferroni method is used to ensure that the familywise error rate is controlled. userOptions.candRDMdifferencesThreshold is interpreted as the familywise error rate, i.e. the probability of getting any false positives under the omnibus null hypothesis that all pairs of candidate RDMs are equally related to the reference RDM.

'none': Do not correct for multiple testing (not recommended). userOptions. candRDMdifferencesThreshold is interpreted as the uncorrected p threshold.

userOptions.nRandomisations

The number of condition-label randomisations (default: 10,000) used to simulate the null distribution that the reference RDM is unrelated to each of the candidate RDMs.

userOptions.nBootstrap

The number of bootstrap resamplings (default: 1,000) used in all selected bootstrap procedures (relatedness test, candidate comparison tests, error bars).

userOptions.plotpValues

This option controls how the significance of the RDM relatedness tests is indicated. If set to '*' (default), then an asterisk is plotted on the bar for each candidate RDM that is significantly related to the reference RDM. Significance depends on the test (see above) and on userOptions. RDMrelatednessThreshold (default: 0.05) and on userOptions. RDMrelatednessMultipleTesting (default: ‘FDR’). Asterisks mark candidate RDMs that are significant at the specified threshold and with the chosen method for accounting for multiple testing. If set to '=', then the uncorrected p value is plotted below the bar for each candidate RDM, and it is plotted in bold type if it is significant by the criteria explained above.

userOptions.barsOrderedByRDMCorr

This option controls the order of the displayed bars (default: true). If set to true, bars corresponding to candidate RDMs are displayed in descending order (from left to right) according to their correlation to the reference RDM. Otherwise, bars are displayed in the order in which the candidate RDMs are passed.

userOptions.figureIndex

This option enables the user to specify the figure numbers for the two created figures (default: [1 2]). The first figure contains the bargraph and the second contains matrices indicating the significance of the pairwise candidate RDM comparisons. The first panel shows the uncorrected-p matrix. The second panel shows the thresholded uncorrected-p matrix. The third panel shows the FDR-thresholded p matrix. The fourth panel shows the Bonferroni-thresholded p matrix.

userOptions.resultsPath

This argument specifies the absolute path in which both figures are to be saved (default: pwd, i.e. current working directory).

userOptions.saveFigurePDF

If set to true (default), the figures are saved in PDF format in userOptions.resultsPath.

userOptions.saveFigurePS

If true (default: false), the figures are saved in post-script format in userOptions.resultsPath.

userOptions.saveFigureFig

If true (default: false), the figures are saved in Matlab .fig format in userOptions.resultsPath.

userOptions.figure1filename

The filename for the bargraph figure, if chosen to be saved (default: 'compareRefRDM2candRDMs_barGraph').

userOptions.figure2filename

The filename for the p-value display figure, if chosen to be saved (default: 'compareRefRDM2candRDMs_RDMcomparisonPvalues').

Return values

stats_p_r

Structure containing numerical statistical results, including effect sizes and p values.

stats_p_r.candRelatedness_r: average correlations to reference RDM

stats_p_r.candRelatedness_p: corresponding uncorrected p values

stats_p_r.SEs: standard errors of average RDM correlations

stats_p_r.candDifferences_r: matrix of bar-height differences (i.e. average RDM-correlation differences)

stats_p_r.candDifferences_p: matrix of p values for all pairwise candidate comparisons

stats_p_r.orderedCandidateRDMnames: candidate RDM names in the order in which the bars are displayed (also the order used for the return values)

stats_p_r.ceiling: ceiling lower and upper bounds

Spearman correlation or Kendall’s τ_A_ for comparing RDMs?

We recommend using Kendall’s τ_A_ whenever categorical models are among the candidate RDMs. The Spearman correlation coefficient favors models that predict tied dissimilarities, as categorical models do. In the presence of noise, the true model (the one that generated the data in a simulation) can be outperformed by a categorical model that gets the major distinctions right (Figure S2), but misses the details.

**<Figure S2 here>**

Estimating the upper bound on the noise ceiling for the RDM correlation

***Pearson correlation.*** When the Pearson correlation is chosen as the measure for comparing RDMs, we z-transform each single-subject RDM. This projects the RDMs onto a hypersphere centered on the origin of RDM space (and restricted to a hyperplane including the origin and orthogonal to the all-1 vector). We refer to the single-subject RDMs on the hypersphere as the “data points”. The z-transform renders the Pearson correlation distance (1-r) proportional to the squared Euclidean distance between points on the hypersphere. This motivates averaging of the z-transformed RDMs, because the average RDM (referred to as the “centroid” below), then, minimizes the sum of squared deviations (across dimensions and subjects), which is the sum of squared Euclidean distances between the centroid and the data points. It obviously also minimizes the *average* of the squared Euclidean distances, which is the sum divided by the number of subjects. However, the centroid will not in general fall on the hypersphere (where squared Euclidean distances are proportional to correlation distances). Since the distances to the data points are smaller for the centroid than for any point on the hypersphere, we can obtain an upper bound by converting the average squared Euclidean distance for the centroid to a correlation. Unfortunately, this bound is not tight. For a tighter bound, we might project the centroid onto the hypersphere first (or equivalently average the correlations between centroid and data points). Although the centroid minimizes the average squared Euclidean distance, we haven’t shown that its projection onto the hypersphere minimizes this quantity among all points on the hypersphere. So the question remains: What RDM strictly maximizes the average correlation to the data points?

An RDM is defined by the direction of a vector in the space. Because we are using the correlation distance, two vectors pointing in the same direction represent identical RDMs, even if their lengths differ. We are seeking the direction that maximizes the average correlation with the data points. The correlations are the cosines of the angles between centroid and data points. Because the data points are on the hypersphere they are all equidistant from the origin. For any given vector, the cosines of the angles to the data points are therefore proportional to the magnitudes of the projections of the data points onto the vector (where the magnitudes are the distances from the origin measured on the vector). The vector maximizing the average correlation is, thus, the vector maximizing the average magnitude of the projections of the data points. The average magnitude of the projections is the magnitude of the projection of the average of the data points. We are, thus, seeking the direction that maximizes the magnitude of the projection of the average RDM. This is the vector representing the average RDM (i.e. the vector emanating from the origin and passing through the average RDM). We therefore obtain a tight upper bound on the noise ceiling by averaging the single-subject RDMs (after z-transform) and computing the average correlation to the single-subject RDMs.

***Spearman correlation.*** For the Spearman correlation, we replace the z-transform used in the context of the Pearson correlation by the rank-transform, and obtain exactly the same results. Like the z-transform, the rank-transform normalizes the mean and the variance of each RDM. Like the z-transform, the rank-transform projects the RDMs onto a hypersphere (within a hyperplane). Rank-transforming each RDM renders the Spearman correlation distance proportional to the squared Euclidean distance. All further results, including the proof of the tight upper bound on the ceiling, also parallel those just described for the Pearson correlation. We therefore compute the average of the rank-transformed single-subject RDMs and compute this RDM’s average Spearman correlation to the single-subject RDMs to obtain a tight upper bound on the noise ceiling.

***Kendall’s τ_A_.*** For Kendall’s τ_A_, the situation is more complicated. However, there again is a space in which the squared Euclidean distance is proportional to the correlation distance. We need to convert each RDM to a vector of pair relations, with one entry for each pair of dissimilarities. The entry is 1 if the first dissimilarity in the pair is larger, and -1 if the second dissimilarity is larger. The squared Euclidean distance between two RDMs in this representation is proportional to the τ_A_ correlation distance (1 - τ_A_). This motivates averaging RDMs in this representation. However, the average in this new embedding space does not correspond to a point representing an RDM. In the present scenario, the embedding space of pair relations is much more complex. The average in this space minimizes the average squared Euclidean distance to the points representing the single-subject RDMs. Because there can be no point in the embedding space having a smaller average squared Euclidean distance to the single-subject RDM points, this provides an upper bound on the noise ceiling for Kendall’s τ_A_. However, the upper bound is not tight at all – presumably because of the much greater complexity of the embedding space. In contrast to the cases of the Pearson and Spearman correlation coefficients described above, we have no closed-form solution for the RDM maximizing the average Kendall-τ_A_ correlation to the single-subject RDMs. We therefore instead average the rank-transformed single-subject RDMs as an initial estimate. We then iteratively optimize this initial estimate by randomly perturbing the dissimilarity values. In practice, this does lead to improvements, although they are very small in our experience, suggesting that the average of the rank-transformed RDMs (which provides the exact solution for the Spearman correlation) might provide an approximate solution for Kendall’s τ_A_. The maximum average Kendall τ_A_ estimated by iterative optimization provides our estimate of the upper bound on the ceiling.

Searchlight RSA

Often one does not know a priori where in the brain a given representation might reside. An analysis based exclusively on predefined ROIs could miss the most important region. To overcome this limitation, searchlight analysis has been proposed as a way of continuously mapping pattern information throughout the volume [2]. Combining the searchlight approach with RSA might enable us to find brain representations that conform to particular stages of processing in computational models. The toolbox supports searchlight RSA.

Similar to ROI-based RSA, the user can test multiple models, by comparing their predicted RDMs to brain RDMs estimated for the local neighborhood around each voxel. A spherical searchlight is centered on each voxel in turn, containing all voxels within a specified radius. An RDM is then computed based on the response patterns of the voxels within the searchlight. The correlation between the searchlight RDM and each model RDM is stored in a map at the central voxel, yielding one searchlight RDM-correlation map per model. To combine searchlight maps across subjects, the maps can be transformed to a common space (e.g. MNI space). Alternatively, the original data can be transformed to the common space as part of the preprocessing of the data. For each model, the searchlight-RDM correlation maps are tested against zero using the Wilcoxon signed-rank test (one-sided) across subjects (subject as random effect). Comparisons between models can similarly be inferentially mapped, by first subtracting the two maps corresponding to the models to be compared within each subject, and then applying the signed-rank test across subjects to the difference maps. To account for multiple testing throughout the search volume, we control the expected false-discovery rate [1].

<Figure S3 here>

The toolbox contains a searchlight demo (DEMO4_RSAsearchlight_sim.m) that implements the subject-random-effects approach. The purpose of this demo is to familiarize the user with the scripts and functions used to compute searchlight maps. The demo simulates a categorical representation restricted to a specific region of the brain. Figure S3 shows the results of the demo with the search volume corresponding to a whole-brain analysis at conventional fMRI resolution (64 by 64 in plane, 32 slices).

An alternative approach to inference would be a fixed-effects randomization test [2-3, 5], which can be applied to single subjects or to groups of less than 12 subjects, where subject random-effects analysis might not be appropriate. Each model RDM would be subjected to condition-label randomization (consistently permuting rows and columns) to simulate the null distribution. Then a group-average searchlight-RDM correlation map would be computed. To this end, we can either compute the correlation between the model RDM and the group-average searchlight RDM, or the average of the correlations between the model RDM and the subject-specific searchlight RDMs. We compute a large number (e.g. 1000) of these null searchlight group maps and store the maximum across the spatial extent of the map each time. We then select the 95th percentile of this null distribution of map maxima as our threshold. We apply this threshold to the searchlight group map computed for the correct labeling of the model RDM (unpermuted).

This procedure accurately controls the familywise error rate and has good sensitivity because the null simulation accounts for the dependencies between nearby locations. When a large volume is expected to contain representational geometries related to the model, controlling the false-discovery rate is expected to provide greater sensitivity than controlling the familywise error rate. False-discovery rate control could be combined with a fixed-effects randomization test by simulating a separate null distribution at every voxel. For a given method of accounting for multiple testing, the fixed-effects approach is expected to be more powerful than the random-effects approach, because the latter attempts to generalize to the population. However, with any continuous mapping method, the multiple testing still reduces the power for a given region, compared to an ROI-based approach that investigates only a small number of regions.

Computing the linear-discriminant *t* value

For each pair of experimental stimuli *i* and *j*, we first seek a weighted average of the response channels that enables us to optimally discriminate *i* and *j*. For homoscedastic multivariate normal errors, the optimal weights are:

*w* = (*p_i_* - *p_j_*) ⋅ Σ^-1^ (Fisher linear discriminant),

where *p_i_* and *p_j_* are the response patterns corresponding to experimental stimuli *i* and *j*, respectively, and Σ is the covariance matrix of the errors (whose height and width equals the number of response channels). We could first estimate single-trial patterns, and then subtract the corresponding condition-mean patterns to obtain the errors matrix for estimating Σ. Alternatively, for time-course data (e.g. fMRI), a linear-model fit to each response channel’s time series could provide the errors matrix (number of time points by number of response channels). Given the errors matrix, we could use the sample covariance or a shrinkage estimator [4] of the covariance. The latter choice promises a more stable covariance estimate that is guaranteed to be invertible. The weights defining the linear discriminant are estimated for dataset 1 (the training data). These weights maximize the *t* statistic (computed after weighted averaging) for contrasting stimuli *i* and *j* in dataset 1. However, because the weights are necessarily somewhat overfitted to dataset 1, the t value from dataset 1 would be positively biased and could not be used to test whether the response patterns contain information discriminating stimuli *i* and *j*. Applying the same weights to the dataset 2 (the test data) and calculating the resulting *t* statistic gives us the linear-discriminant *t* (LD-*t*) value. The LD-*t* is the t value for dataset 2 computed after projection onto the linear discriminant estimated with data set 1. It is a valid t value (t distributed under the null hypothesis of equal response pattern distributions for stimuli *i* and *j*) and can be used to test discriminability of *i* and *j*.

The LD-*t* value is a crossvalidated measure of the discriminability of the two stimuli. Low discriminability could be due to similar responses to the two stimuli or high levels of noise. One can think of the LD-*t* as a crossvalidated variation on the Mahalanobis distance. The mathematical relationship between the Mahalanobis distance and the Fisher linear discriminant contrast (whose division by its standard error yields the LD-*t*) is clarified in Figure S4.

**<Figure S4 here>**

For full crossvalidation, we can exchange the two datasets, using dataset 2 for training and dataset 1 for computing the *t* value. We can then average the two *t* values to get a more stable estimate. Although datasets 1 and 2 are independent, the two directions (folds of crossvalidation) are not independent. The average of the *t* values therefore has a standard error which is smaller than 1 (the standard error of a proper *t* value), but larger than $1/\sqrt{2}$. In other words, the average *t* is more stable (lower standard error) than a *t* value, but we don’t know exactly by what factor. Converting it to a *p* value for inference provides a conservative test for pattern information discriminating individual pairs of stimuli in single subjects.

We can assemble the LD-*t* values for all pairs of stimuli in an LD-*t* RDM. This RDM could contain the LD-*t* for the two directions in symmetric positions. However, we will only consider one entry per pair of stimuli (lower-triangular vector of the RDM) and define each entry as the average LD-*t* across the two directions. We can convert this average LD-*t* RDM to a p matrix and control the false-discovery rate or the familywise error rate (Bonferroni) to account for multiple comparisons across pairs of stimuli. This provides a sensitive method for testing many pairs of stimuli for discriminability based on little data per stimulus.

We could consider alternative techniques such as the linear support vector machine (SVM) for estimating the discriminant dimension. Note, however, that a linear SVM discriminant would reduce to the difference vector between *p_i_* and *p*_j_, unless we have multiple pattern estimates per stimulus. The Fisher linear discriminant is attractive in this context, because it characterizes the shape of the error distribution by a multivariate Gaussian, using a pooled covariance estimate. This approach is particularly attractive for fMRI data, where the errors are usually assumed to be homoscedastic and known to be correlated.

**<Figure S5 here>**

For group analysis, we can average the LD-*t* RDMs and then multiply the average LD-*t* values by $\sqrt{n}$, where $n$ is the number of subjects. Fixed-effects inference can then be performed as for the single-subject LD-*t* RDM by converting the *t* values to *p* values. Alternatively, we can perform random-effects inference taking advantage of the distribution across subjects (if there are 12 or more). We could use either a single-sample *t* test or the Wilcoxon signed-rank test [6] to test, for each pair of stimuli, if the average LD-*t* is greater than 0. Figure S5 shows the average LD-*t* RDM for 20 simulated subjects and the results of random-effects inference. The LD-*t* RDM reveals the simulated structure. It provides an interesting alternative to the other pattern dissimilarity measures.

References

1. Benjamini Y, Hochberg Y, (1995). Controlling the false discovery rate: a practical and powerful approach to multiple testing. Journal of the Royal Statistical Society. Series B (Methodological), 289-300.
2. Kriegeskorte N, Goebel R, Bandettini P, (2006). Information-based functional brain mapping. Proceedings of the National Academy of Sciences of the United States of America 103: 3863–3868.
3. Kriegeskorte N, Mur M, Bandettini P, (2008). Representational similarity analysis–connecting the branches of systems neuroscience. Frontiers in systems neuroscience 2.
4. Ledoit O, & Wolf M (2003). Improved estimation of the covariance matrix of stock returns with an application to portfolio selection. Journal of Empirical Finance, 10(5): 603-621.
5. Nichols TE & Holmes AP (2002). Nonparametric permutation tests for functional neuroimaging: a primer with examples. Human brain mapping, 15(1): 1-25.
6. Wilcoxon, F, (1945). Individual Comparisons by Ranking Methods, Biometrics Bulletin. Vol1, No 60: 80-83.

Figure Legends

**Figure S1: Decision process for selection of statistical tests.** The flow diagram above shows the default decision process by which the statistical inference procedures are chosen in the toolbox. The analyses in Figures 4 and 5 of the paper correspond to paths in the flowchart that lead to the leftmost (simulation in Figure 4) and second from right (real data in Figure 5) box at the bottom. Note that the flowchart does not capture all possibilities. For example, the fixed-effects condition-label randomization test of RDM relatedness can be explicitly requested, even when there are 12 or more subjects’ estimates of the reference RDM and the random-effects signed-rank test would be chosen by default.

**Figure S2: Spearman versus Kendall’s τ_A_ rank correlation for comparing RDMs.** Here the inferential results from the paper using Kendall’s τ_A_ (Figures 4, 5) are presented again (panels A, B), and compared to the results obtained using the Spearman correlation (panels C, D). The two rank correlation coefficients differ in the way they treat categorical models (blue bars) that predict tied dissimilarities. **(A)** For the simulated data, Kendall’s τ_A_ correctly reveals that the true model (red bar) best explains the data. It is the only model that reaches the ceiling range, and it outperforms every other candidate significantly (horizontal lines above the bars). **(C)** For the Spearman correlation, the true model no longer has the greatest average correlation to the reference RDM. Two categorical candidate RDMs appear to outperform the true model, and significantly so (horizontal lines). Both of these categorical models and the true model now fall in the ceiling range. **(B, D)** For the real data, as well, categorical models (blue) are favored by the Spearman correlation.

**Figure S3: Group-level results for 20 simulated subjects.** **(A)** A representational geometry of 64 patterns falling into two clusters was simulated in a brain region (shown in green) in each of 20 subjects. Data outside the green region was spatially and temporally correlated noise (typical of fMRI data) with no design-related effects. Searchlight maps (searchlight radius = 7 mm) were generated by computing the correlation between a model RDM (reflecting the true cluster structure of the simulated patterns) and the searchlight RDM at each voxel in each subject. **(B)** At each voxel, a one-sided signed-rank test was applied to the subject-specific correlation values. The 3D map of p values was thresholded so as to control the expected false-discovery rate at 0.05. Voxels exceeding the threshold are highlighted (yellow). The maps in both panels are superimposed on an anatomical T1 image re-sliced to fit the simulated brain dimensions. The red contours depict the borders of the brain mask. RDMs were computed for searchlights centered on each voxel within the brain mask.

**Figure S4: Relationship between the linear-discriminant *t* value and the Mahalanobis distance.** In the Mahalanobis distance, the inverse of the error covariance (Σ) is pre- and post-multiplied by the difference vector between the pattern estimates (p1 and p2). If we use pattern estimates from an independent dataset (dataset 2) for the post-multiplication, we obtain the dataset-2 contrast estimate on the Fisher linear discriminant fit with dataset 1. This is because the first part of the definition of the Mahalanobis distance equals the weight vector w of the Fisher linear discriminant. The LD-*t* is the Fisher linear discriminant contrast (as shown) normalized by its standard error (estimated from the residuals of dataset 2 after projection on the discriminant dimension).

**Figure S5: Random-effects inference on LD-*t* RDMs. (A)** Two fMRI datasets were simulated for 20 subjects. We simulated fMRI time-course data Y based on a realistic fMRI design matrix (X) with hemodynamic response predictors for 64 stimuli and patterns (B) with a predefined hierarchical cluster structure (two categories, each comprising two subcategories). The simulated data were Y=XB+E, where E is the time-by-response errors matrix, consisting of Gaussian noise temporally and spatially smoothed by convolution with Gaussians to create realistic degrees of temporal and spatial autocorrelation. The LD-*t* RDMs were computed for each subject and averaged across subjects. The group-average LD-*t* RDM is shown using a percentile color code. **(B)** Inference on LD-*t* RDMs with subject as random effect. LD-*t* analysis can serve the same purpose as classifier decoding analysis, to test for pattern information discriminating two stimuli. For each pair of stimuli, we used a one-sided signed-rank test across subjects and obtained a *p* value. The left panel shows the pairs with p < 0.05, uncorrected (red). The middle panel shows the pairs that survive control of the expected false-discovery rate (q < 0.05). The right panel shows the pairs that survive Bonferroni correction (p < 0.05, corrected).

**Software S1.** The zip file contains the complete RSA toolbox. It also contains demo functions and brain-activity- and behavior-based representational dissimilarity matrices used by the demo functions. DEMO1_RSA_ROI_simulatedAndRealData.m reproduces the main parts of figures 2-5 of the main paper. The toolbox is written in Matlab and requires the Matlab programming environment.
